# A Phytotoxin with Selective Herbicidal Activity and Related Metabolites from the Phytopathogenic Fungus *Bipolaris cookei* SYBL03

**DOI:** 10.3390/molecules29133040

**Published:** 2024-06-26

**Authors:** Haiyan Li, Jingzhuo Hou, Bing Li, Lizhong Zhang, Zhiguo Yu

**Affiliations:** 1 College of Plant Protection, Shenyang Agricultural University, Shenyang 110866, China; lihaiyan98@syuct.edu.cn; 2 College of Environmental and Safety Engineering, Shenyang University of Chemical Technology, Shenyang 110142, China; z2023538@stu.syuct.edu.cn (J.H.); libinglucky@163.com (B.L.); 3 College of Computer Science and Technology, Shenyang University of Chemical Technology, Shenyang 110142, China; zhanglz_2009@syuct.edu.cn

**Keywords:** *Bipolaris cookei*, secondary metabolites, herbicidal activity, concentration-dependent dual effect

## Abstract

Weeds are a serious threat to crop production, and the utilization of secondary metabolites of phytopathogenic fungi is considered to be an effective method of weed control. In this study, eight compounds were isolated and purified from the mycelium and fermentation broth extracts of *Bipolaris cookei* SYBL03. The compounds (**1**–**8**), except **2** and **6**, are reported for the first time from this genus. The herbicidal activities of compounds **1**–**8** were studied by evaluating their effects on the seed germination and seedling growth of monocotyledonous and dicotyledonous weeds. The results indicated that compound **7** (*Cyclo*-*N*-methylphenylalanyltryptophenyl, cNMPT) exhibited a concentration-dependent dual effect on the growth of weed seedlings and selective herbicidal activity against dicotyledonous weeds. We further investigated the morphological and physiological responses of roots of *Amaranthus retroflexus*, a dicotyledonous weed, to compound **7**. Some changes were found in seedlings grown in 400 μg/mL compound **7** solution for 96 h, such as shortening and swelling of elongation zone cells, reduced number and length of root hairs, damage and wrinkling of the root surface, occurrence of electrolyte leakage, and an increase in ethylene content. These results suggest that compound **7** may exert herbicidal activity by causing stress to weed seedlings. Increased ethylene production could be involved in the response of plants to compound **7**.

## 1. Introduction

It is estimated that the global population will reach 9.7 billion by 2050 [1], leading to increased demand for food production. According to the Food and Agriculture Organization of the United Nations (FAO), at least a 50% increase in agricultural food production will be needed to meet the demand [2]. In agricultural production, various factors like animal pests, plant pathogens, and weeds can impact on grain yield, and weeds are responsible for the highest potential loss (34%) [3]. Currently, herbicides are widely used as the most economical method for controlling weeds all over the world [4].

Herbicides are employed to effectively manage and eliminate weeds, with the goal of protecting arable land, improving crop productivity, and promoting agricultural sustainable development [5]. Additionally, they can also reduce labor and machinery costs and increase profits. Nevertheless, the prevalent utilization of chemical herbicides leads to significant problems, including ecological degradation, soil pollution, destruction of beneficial microorganisms, pesticide resistance, and contamination of agricultural products, posing substantial health risks to humans and animals [6]. Consequently, there is an immediate necessity to explore newer environmentally friendly herbicides from natural resources, including metabolites from animals, plants, and microbes [7].

Many plant pathogens have been researched and identified as potential agents for controlling weeds in various arable crops [8]. Research indicates that the action of various phytopathogenic fungi is attributed to secondary metabolites, including terpenoids, polyketides, nonribosomal peptides, and compounds produced from shikimic acid [9]. Many of them are non-host-specific and have inhibitory effects, not only on host plants but also on others [10], so they have the potential to be developed into natural herbicides [11]. So far, several non-host-specific phytotoxins have been afforded by the genus *Bipolaris* [12], such as cochlioquinone derivatives [13,14], prehelminthosporol, dihydroprehelminthosporol, victoxinine, and prehelminthosporolactone [15].

*Bipolaris cookei* is a necrotrophic fungus that causes a sorghum disease called target leaf spot, and it can give rise to a 50% reduction in production during a serious outbreak [16,17]. The current research on the *B. cookei* mainly focuses on the pathogenic mechanism, gene sequence determination, and the process of host cell invasion [16,17,18]. *B. cookei* not only infects sorghum but also other species, such as rice [19], wheat [20], and maize [21]. Winder and Dyke [22] prepared a solution of *B. cookei* (=*B. sorghicola*) (isolated BS1) spores and sprayed it on two-week-old Johnson grass. Seven days after the inoculation, BS1 killed 100% of the Johnson grass. This report suggests that *B. cookei* may produce potent phytotoxic metabolites. In 1987, Sugawara et al. [23] isolated six ophiobolin compounds from *B. cookei* (=*Drechslera sorghicola*). These were all capable of causing brownish lesions in corn, Johnson grass, and sorghum. In 1989, Pena-Rodriguez and Chilton [24] isolated four ophiobolin compounds from the ethyl acetate (EtOAc) extract of the fermentation broth of *B. cookei* (=*B. sorghicola*). The lesions caused by these compounds on maize (*Zea mays*), bentgrass (*Agrostis alba*), sicklepod (*Cassia obtusifolia*), and morning glory (*Ipomoea nil*) were all necrotic spots similar to those found on sorghum. These results indicate that *B. cookei* has the ability to produce non-host-specific toxins for weed control. However, there has been no further research on the toxins produced by this plant pathogenic fungus except ophiobolin compounds.

To discover more structurally intriguing and bioactive metabolites from this fungus, we isolated and purified the EtOAc extracts from broth and mycelia of *B. cookei* SYBL03. And then, we selected six common weed species from different genera in Northeast China as targets to evaluate the herbicidal activity of the isolated compounds.

## 2. Results

### 2.1. Isolation, Purification, and Structure Elucidation

**Figure 1:**
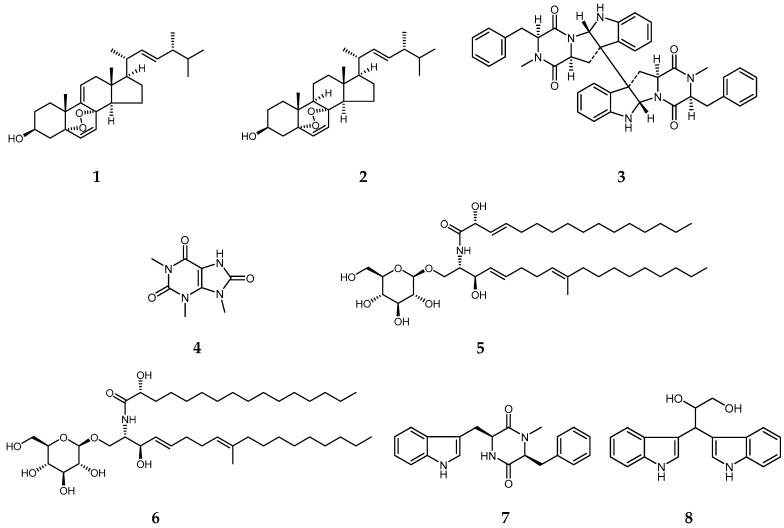
Chemical structures of compounds **1**–**8** isolated from *B. cookei*.

The EtOAc extracts of mycelia and fermentation supernatant of *B. cookei* SYBL03 were subjected to column chromatography (CC) on silica gel to provide six (Fr. A–F) and five fractions (Fr. A′–E′), respectively. The fractions were further separated by CC over silica gel, Sephadex LH-20 and semi-preparative HPLC, and recrystallization to afford eight compounds. Their structures were identified as 9,11-dehydroergosterol peroxide (=5α,8α-epidioxyergosta-6,9(11),22-trien-3β-ol) (**1**) [25], ergosterol peroxide (=5α,8α-epidioxyergosta-6,22-dien-3β-ol) (**2**) [25], (–)-ditryptophenaline (**3**) [26,27], 1,3,9-trimethyluric acid (**4**), cerebroside A (**5**) [28], cerebroside B (**6**) [29], *cyclo*-*N*-methylphenylalanyltryptophenyl (**7**) [26], and (2*S*)-3-di-1*H*-indol-3-yl-1,2-propanediol (**8**) [30] (Figure 1), based on their spectroscopic data (Figures S1–S25, Supplementary Materials) and compared with reported values in the previous literature.

According to the ^1^H and ^13^C NMR data, the structure of compound **4** was very similar to that of 8-oxocaffeine [31]. But the analysis of the HMBC correlations allowed for the position of three methyl groups at N-1, N-3 and N-9, respectively (Figure S12).

### 2.2. Phytotoxic Activity of Compounds **1**–**8**

**Figure 2:**
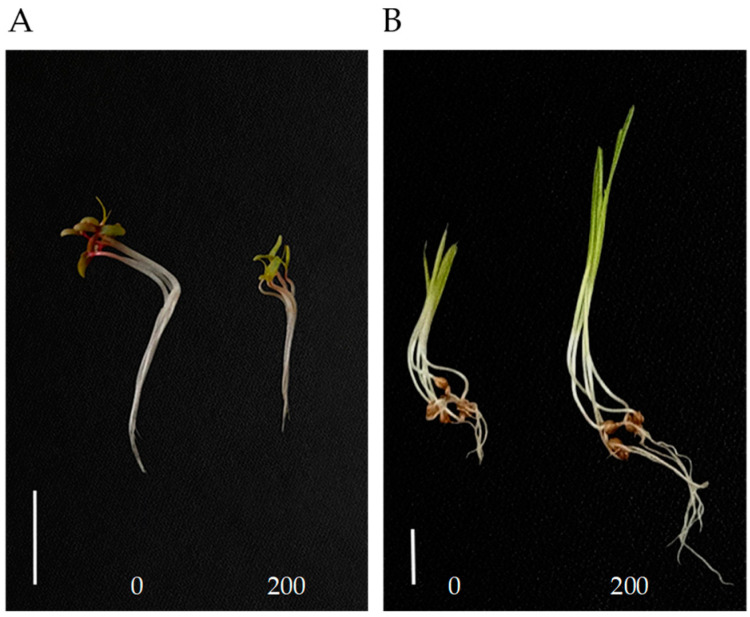
*A. retroflexus* (**A**) and *E. crus-galli* (**B**) seedlings grown with 0 (control) and 200 μg/mL compound **7** for 96 h. Scale bar = 1 cm.

We investigated the effects of eight compounds at 200 μg/mL on the seedling growth of *Amaranthus retroflexus* and *Echinochloa crus-galli*. Compounds **3**–**6** and **8** showed no phytotoxicity to the seedling growth of two weeds. Compound **1** had weaker inhibitory activity against the seedling growth of *A. retroflexus*. Compounds **2** and **7** had obvious inhibitory effects on *A. retroflexus*, with inhibition rates of 39% and 46% against shoot growth, and 56% and 52% against root growth, respectively (Table 1 and Figure 2A). Interestingly, compound **7** significantly promoted the growth of *E. crus-galli*; the shoot and root length increased by 46% and 135% as compared with control, respectively (Table 1 and Figure 2B). These results indicated that the effects of compound **7** on seedling growth may be different between monocots and dicots.

### 2.3. Effect of Compound **7** on the seedling growth of Monocots and Dicots

**Figure 3:**
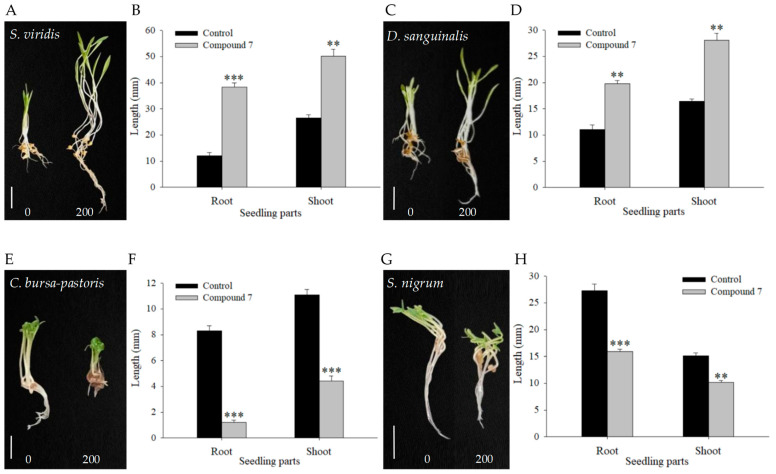
Effect of compound **7** on the seedling growth of monocots (**A**–**D**) and dicots (**E**–**H**). Seedlings were grown with 0 (control) and 200 μg/mL compound **7** for 96 h for all except *C. bursa-pastoris* (144 h). The growth of *S. viridis* (**A**,**B**) and *D. sanguinalis* (**C**,**D**) was promoted, while that of *C. bursa-pastoris* (**E**,**F**) and *S. nigrum* (**G**,**H**) was inhibited by compound **7**. Data presented as mean ± SE, and asterisks indicate a significant difference (two-tailed Student’s *t*-test, ** *p* < 0.01 and *** *p* < 0.001) as compared with the control group. Scale bar = 1 cm.

The following experiment aims to further ascertain whether the different effects of compound **7** on monocots and dicots are universal. Two dicots (*Solanum nigrum*, *Capsella bursa-pastoris*) and two monocots (*Setaria viridis*, *Digitaria sanguinalis*) were selected as test weeds for the bioassay to estimate the influence of compound **7** on seedling growth at 200 μg/mL. The results showed that compound **7** had a growth-promoting effect on monocots. At 96 h after treatment, the shoot and root length of *S. viridis* increased by 89% and 216% (Figure 3A,B), and those of *D. sanguinalis* increased by 71% and 80% compared with the corresponding controls, respectively (Figure 3C,D). However, compound **7** showed a growth inhibitory effect on the seedling growth of dicots. The shoot and root lengths of *C. bursa-pastoris* were inhibited by 60% and 86% at 144 h after treatment, and those of *S. nigrum* inhibited by 32% and 42% at 96 h following treatment compared with the corresponding controls, respectively (Figure 3E–H). To sum up, compound **7** had different effects on monocots and dicots, promoting the growth of monocots and inhibiting the growth of dicots at a certain concentration.

### 2.4. Effect of Compound **7** at Different Concentrations on Dicotyledons

**Figure 4:**
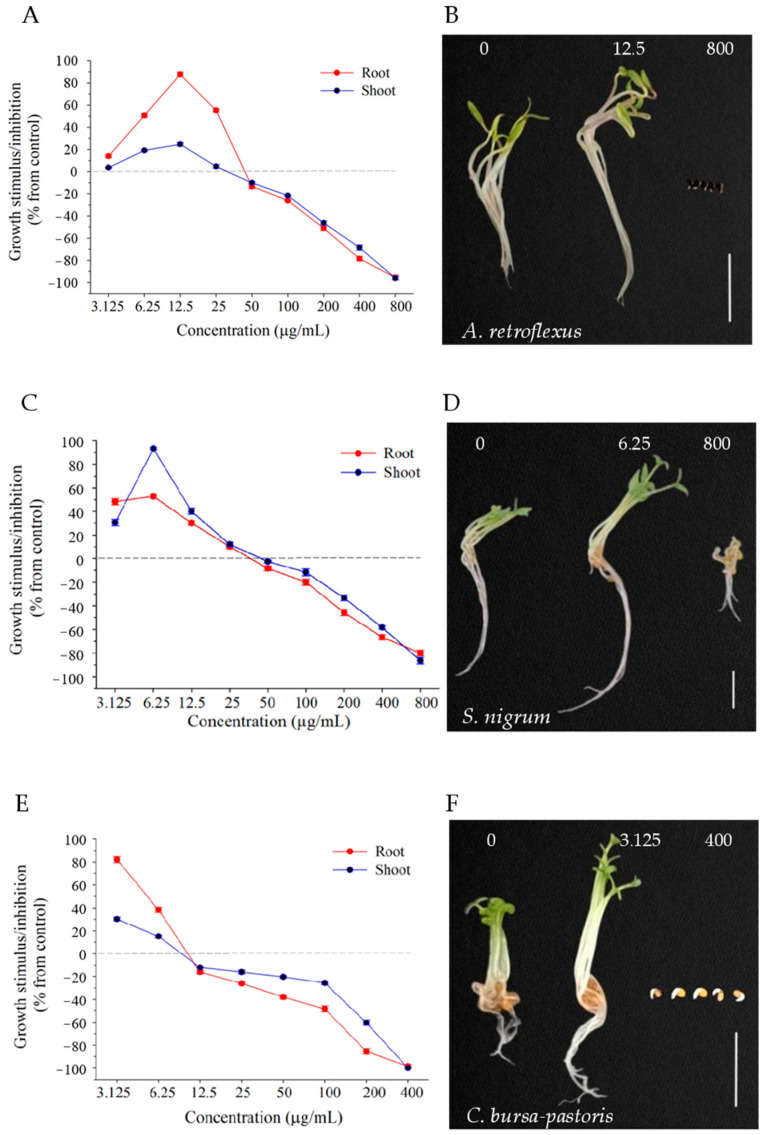
Effect of compound **7** at different concentrations on the seedling growth of three dicotyledonous weeds, *A. retroflexus* (**A**), *S. nigrum* (**C**) and *C. bursa-pastoris* (**E**). The photographs show the optimal promotion and inhibition effects of compound **7** on *A. retroflexus* (**B**), *S. nigrum* (**D**) and *C. bursa-pastoris* (**F**), respectively. Scale bar = 5 mm.

We further investigated the effect of compound **7** on the growth of dicotyledonous weeds (*A. retroflexus*, *S. nigrum* and *C. bursa-pastoris*) in a relatively wide concentration range (3.25–800 µg/mL). With increased treatment concentration, the root and shoot growth of three dicotyledonous weeds changed from promotion to inhibition compared with the control group (Figure 4A,C,E). Compound **7** at lower concentrations enhanced the growth of *A. retroflexus* (≤25 μg/mL)*, S. nigrum* (≤25 μg/mL), and *C. bursa-pastoris* (≤6.25 μg/mL). These concentrations were significantly lower than the growth-promoting concentration (200 μg/mL) of three monocotyledonous weeds. The maximum growth promotion effects on *A. retroflexus*, *S. nigrum,* and *C. bursa-pastoris* seedlings were observed at concentrations of 12.5, 6.25, and 3.125 μg/mL, respectively. At the optimal growth-promoting concentration, the growth of roots and shoots separately increased by 88% and 25% for *A. retroflexus* (Figure 4A)*,* 53%, and 93% for *S. nigrum* (Figure 4C)*,* and 82% and 30% for *C. bursa-pastoris* (Figure 4E) compared with control. At a relatively high concentration, these seedlings began to exhibit concentration-dependent growth inhibition. At the highest treatment concentration (800 μg/mL), the seedling growth of *A. retroflexus* and *C. bursa-pastoris* almost completely stopped (Figure 4A,E), and the root and shoot growth of *S. nigrum* seedlings reduced by 80% and 86% compared with control (Figure 4C), respectively. The optimal promotion and inhibition effects of compound **7** on the three dicotyledons are shown in Figure 4B,D,F. At moderate inhibitory concentrations, compound **7** showed stronger inhibition against roots than shoots for the three dicotyledonous weeds, suggesting that roots are more susceptible to compound **7** than shoots. The results indicate that the effect of compound **7** on plants depends on its concentration, which promotes growth at low concentrations, inhibits growth at high concentrations, and completely blocks growth at higher concentrations.

### 2.5. Effect of Compound **7** on Seed Germination

**Figure 5:**
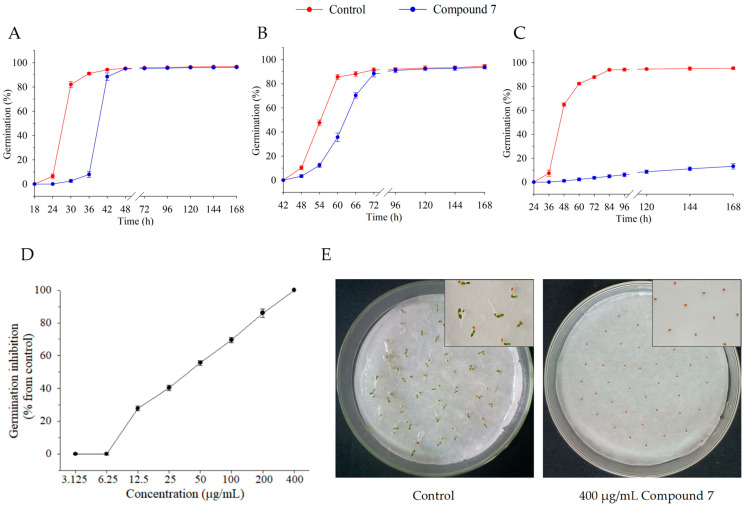
Effect of compound **7** on seed germination. (**A**–**C**) The inhibition of compound **7** at 200 µg/mL against seed-germination process of three dicots, *A. retroflexus* (**A**)*, S. nigrum* (**B**)*,* and *C. bursa-pastoris* (**C**). (**D**) Compound **7** dose-response curve of *C. bursa-pastoris* gemination inhibition after 7 d; (**E**) Germination inhibition of compound **7** against *C. bursa-pastoris* at 400 µg/mL for 7 d.

An investigation was conducted to examine the effect of compound **7** on seed germination at 200 µg/mL. The results showed that the germination process and rate of the monocots (*S. viridis*, *D. sanguinalis* and *E. crus-galli*) were not significantly affected. In contrast, among the dicots (*A. retroflexus*, *S. nigrum*, and *C. bursa-pastoris*), the seed germination of *A. retroflexus* and *S. nigrum* was delayed, although the germination rate after **7** d remained unaffected (Figure 5A,B). However, the germination of *C. bursa-pastoris* seeds was significantly inhibited (Figure 5C).

Therefore, we further investigated the effects of different concentrations (3.125–400 µg/mL) of compound **7** on the seed germination of *C. bursa-pastoris*. The results showed that the seed germination of *C. bursa-pastoris* was inhibited by compound **7** in a concentration-dependent manner at concentrations greater than 6.25 µg/mL, and completely inhibited at a concentration of 400 µg/mL (Figure 5D,E).

### 2.6. Inhibitory Mechanism of Compound **7** on A. retroflexus Root Growth

**Figure 6:**
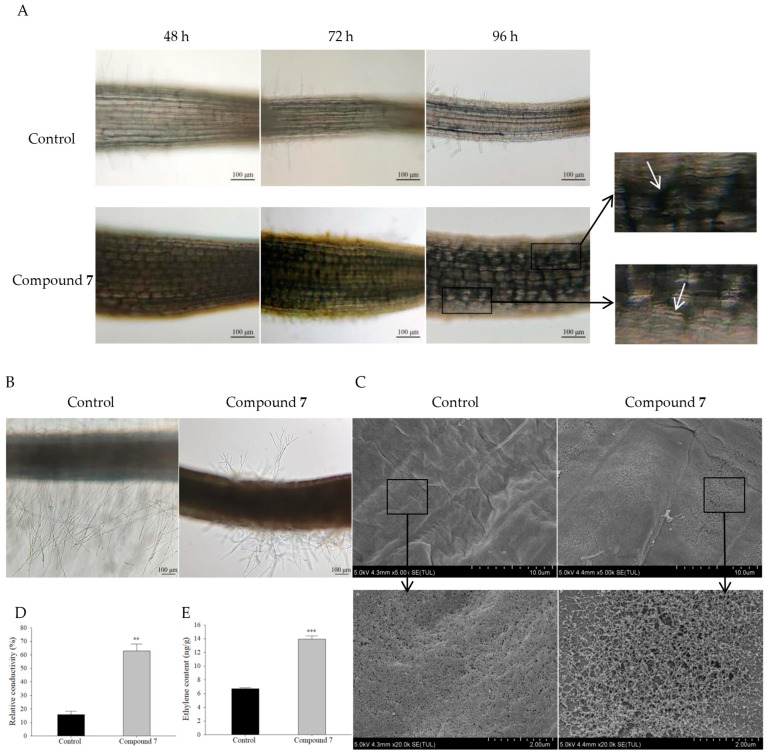
Phenotypic, cell morphological and physiological effects of compound **7** on *A. retroflexu* root at 400 μg/mL. (**A**) Root phenotype and cell shape of the elongation zone. White arrows in the picture showed damaged and wrinkled cell surfaces under the light microscope; (**B**) The morphology of root hairs; (**C**) The surface morphology of roots under SEM. Seedlings were grown for 96 h, and the root elongation zone was observed; (**D**) Root conductivity at 96 h following application of compound **7**. (**E**) Ethylene content in roots at 96 h following application of compound **7**. Data presented as mean ± SE, and asterisks indicate a significant difference (two-tailed Student’s *t*-test, ** *p* < 0.01 and *** *p* < 0.001) as compared with the control group.

In order to better understand the inhibitory capacity of compound **7**, we further investigated the morphological and physiological effects of compound **7** on *A. retroflexus* roots. *A. retroflexus* seedlings grown in 400 μg/mL of compound **7** solution for 48, 72, and 96 h were observed using an optical microscope. It is clear that compound **7** caused the radial swelling in the root phenotype and a reduction in cell length in the root elongation zone compared with control (Figure 6A). We further observed the radial expansion of the treated cells under scanning electron microscope (SEM) (Figure 6C). In addition, the SEM images revealed the damage effects of compound **7** on the elongation-zone cells, which were visible as cracks/fractures, breakages, and wrinkles relative to an indistinct, smooth/even, and unbroken shape, as shown in the control root surface (Figure 6A,C). Consistent with this finding, the relative conductivity of the seedlings grown in 400 μg/mL compound **7** for 96 h was 62.84%, which was much higher than that of the control (15.76%) (Figure 6D). These results indicate that the cell membrane can be damaged by compound **7**, leading to the release of intracellular electrolytes.

Additionally, we found that compound **7** delayed the root hair initiation and significantly inhibited root hair growth (Figure 6A,B). In the control group, the development of root hairs entered the elongation stage at 48 h after sowing, but in the experimental group, the root hair cell wall began to bulge out by 72 h (Figure 6A). Notably, branched root hairs were found in the treatment group (Figure 6B). This finding was similar to previous reports in which branched root hairs will form under abiotic stress or nutrient deficiency [32,33]. These results suggest that the delayed and abnormal development of root hairs may be due to the effects of stress caused by compound **7**.

In plants, ethylene production often enhances the tolerance to given environmental conditions [34]. Here, the ethylene content in *A. retroflexus* roots treated with compound **7** for 96 h was doubled compared with the control group (Figure 6E), which may be associated with the inhibition of root growth, the delayed development of root hairs, and the stress response.

## 3. Discussion

The utilization of fungal phytotoxins for the generation of environmentally benign bioherbicides is undergoing a period of accelerated development [10,11,35]. In this study, eight compounds were isolated and purified from the mycelium and fermentation broth extracts of *B. cookei* SYBL03, an important phytopathogenic fungus causing sorghum leaf diseases. The compounds (**1**–**8**), except **2** and **6,** are reported for the first time from this genus. Compound **1** was first isolated in 1983 from the mycelium of *Guignardia laricina* by Otomo et al. [36] and showed potential anti-inflammatory and antitumor activities [37]. In our study, compound **1** exhibited weaker inhibitory activity against the seedling growth of *A. retroflexus* (Table 1). Compound **3**, initially isolated in 1977 from the secondary metabolites of *A. flavus* by Springer et al. [38], demonstrated significant analgesic and anti-inflammatory activities [27]. Compound **4** displayed antioxidant activity. Although this structure has been synthesized before, NMR data from a natural source have not been reported. Compound **5**, obtained for the first time in 1987 from the cell extract of *Pachyhasium* sp. by Sitrin et al. [39], has exhibited a variety of biological activities, including anti-inflammatory activity [40], analgesia [41], plant defense induction [42], and nematicidal activities against *Bursaphelenchus xylophilus* [43], highlighting its application prospects in agriculture and medicine. Compound **6**, first isolated in 1983 from *Schizophyllum commune* by Kawai and Ikeda [44], has showed neuroprotection [45], analgesia [41], and plant resistance induction [46]. Compound **8**, isolated for the first time in 1977 from *Balansia epichloe* (Weese) by Porter et al. [47], exhibited antibacterial activity against *Clostridium perfringens* [48]. In the present study, compounds **3**–**6** and **8** did not show any remarkable phytotoxicity to *A. retroflexus* and *E. crus-galli.*

Here, only compounds **2** and **7** showed obvious inhibitory effects on *A. retroflexus.* Compound **2**, isolated for the first time in 1947 from the mycelium of *Aspergillus fumigatus* by Wieland and Prelog [49], has exhibited a wide range of biomedical activities, including antiviral [50], antitumor [51], anti-inflammatory [52], immunomodulatory [50], antifungal, and cytotoxic [53] activities. In addition, Macías et al. [54] reported its phytotoxicity against the seedling growth of *E. crus-galli*. In contrast, we found that compound **2** had no significant effect on the seedling growth of *E. crus-galli*. This inconsistency may be due to the differences in treatment dosage and culture conditions. It is noteworthy that compound **7** exhibited preferable selective herbicidal activity against dicotyledonous weeds. Compound **7** is a cyclic dipeptide compound, which was first separated from the culture filtrate of *Aspergillus* sp. by Luo et al. [55] and showed no obvious effect on the growth of bacteria and nematodes. Subsequently, Ma et al. [26] demonstrated that 400 µg/mL of compound **7** is an effective inhibitor of seed germination in *A. thaliana*. Similarly, our study also revealed that compound **7** at a concentration of 400 μg/mL inhibited the seed germination of *C. bursa-pastoris* by 100% (Figure 5A). Furthermore, our findings indicated that it significantly affected the seedling growth of *A. retroflexus*, *S. nigrum,* and *C. bursa-pastoris*.

Further research demonstrated that the effect of compound **7** on the growth of weed seedlings exhibited a concentration-dependent dual role. At low concentrations, the treatment promoted growth, whereas with an increasing concentration, growth was inhibited (Figure 4). It was found that the monocots and dicots exhibited differential sensitivity to compound **7** (Figure 2 and Figure 3), with seedling growth promoted in monocots (the increase ranging from 46% to 216% in shoot and root growth of *S. viridis*, *D. sanguinalis* and *E. crus-galli*) while inhibited in dicots (the decrease ranging from 32% to 86% in shoot and root growth of *A. retroflexus*, *C. bursa-pastoris* and *S. nigrum*) at a concentration of 200 μg/mL. The difference in sensitivity to plant toxic compounds between monocots and dicots has been widely reported in auxin herbicides [56,57,58]. Auxin herbicides exert selective action, with a preference for dicotyledonous weeds in cereal crops [59]. These findings showed that the effect of compound **7** on weeds might be comparable to that of auxin herbicides.

Based on the herbicidal activity of compound **7**, we further investigated its mechanism of growth inhibition. The results showed that the application of compound **7** inhibited the root elongation and caused shortening and swelling of elongation-zone cells of *A. retroflexus*, which led to a swollen root phenotype (Figure 6A,C). This is similar to what DeBolt et al. [60] observed in *A. thaliana* seedlings, where the cells expanded manifold in diameter relative to controls, resulting in swollen roots.

Root hairs are long tubular-shaped outgrowths from root epidermal cells [61]. In *Arabidopsis thaliana*, the mechanism of the root epidermis is based on the positional relationship between epidermal cells and underlying cortical cells. The cells located outside an anticlinal cortical cell wall will undergo three processes: change in root hair cell fate, root hair initiation, and root hair elongation, leading to the development of mature root hairs [62]. Here, the root hair initiation was delayed, and the growth of root hairs was inhibited in the treatment group, showing a reduction in number and shortening in length (Figure 6A,B). In previous studies, herbicides have been found to inhibit the growth of plant root hairs. Zhou et al. [63] treated maize with imazethapyr, which impaired root hair growth, showing significantly sparser and shorter root hairs. Interestingly, it was observed that the branching of root hairs occurred, which may be attributed to the influence of stress (Figure 6B). Previous work revealed that branched root hairs will form under abiotic stress or nutrient deficiency [32,33]. In addition, electrolyte leakage and cell death accompany plant response to stresses, such as drought, salt stress, heavy metal contamination, extreme temperatures, and compounds with herbicidal activity [64,65]. Some natural phytotoxins, such as cercosporin and syringomycin, cause electrolyte leakage through both light-dependent and non-light-dependent mechanisms, ultimately resulting in plant death [66]. In our study, microscopic observation showed the cracks/fractures, breakages, and wrinkles on the root surface (Figure 6A,C), and, meanwhile, we also detected the occurrence of electrolyte leakage (Figure 6D), which further suggests that the roots were subjected to drug stress.

Previous work has shown that the plant hormone ethylene is involved in plant growth and development [67]. Ethylene also plays a crucial role in plant response or adaptation to biotic and abiotic stress [34]. In our study, the phytohormone detection revealed an increase in ethylene content from 6.7 to 13.9 ng/g. This finding is in accordance with those previously reported. Excessive stimulation of ethylene production through the induction of 1-aminocyclopropane-1-carboxylic acid (ACC) synthase in biosynthesis is a well-known early event of auxin herbicides [57,58,67]. Ethylene treatment induced root shortening and swelling in *Arabidopsis* seedlings [68]. These results indicate that ethylene may be involved in the root growth inhibition of *A. retroflexus* seedlings induced by compound **7**.

This is the first report showing that compound **7** has the selective effect against monocotyledonous and dicotyledonous weeds. This selectivity may be related to the differential sensitivity of monocots and dicots to compound **7**. Monocotyledonous weeds are less sensitive to compound **7** than dicotyledonous weeds. The herbicidal activity may be associated with the compound **7**-induced stress. Increased ethylene production could be involved in the response of plants to compound **7**. There should be further investigations to elucidate the mechanism of compound **7** on plants, with a particular focus on the proteomic, transcriptomic, and biochemical analyses.

## 4. Materials and Methods

### 4.1. General Experimental Procedures

Optical rotation was obtained on a JH-P100 digital automatic polarimeter (Shanghai Jiahang Instruments Co., Ltd., Shanghai, China) at 20 °C. The ^1^H- and ^13^C-NMR spectra were recorded on a Bruker Avance III 600 MHz NMR spectrometer using the solvent peak as an internal standard (Bruker BioSpin, Rheinstetten, Germany). The HMBC experiment was recorded using the pulsed-field gradients. Electrospray ionization mass spectra (ESI-MS) were performed on Waters ACQUITY RDa mass detector (Waters Co., Milford, MA, USA). The Amberlite XAD-16N resin (560–710 µm, DuPont, Wilmington, DE, USA) was used for isolating and concentrating organic compounds from fermentation supernatant. Column chromatography was performed using silica gel (100–200 and 200–300 mesh, Qingdao Haiyang Chemical Co., Ltd., Qingdao, China) and Sephadex LH-20 (25–100 µm, GE Healthcare, Uppsala, Sweden). Analytical and preparative thin-layer chromatography (TLC and PTLC) was carried out on pre-coated silica gel GF_254_ plates (0.25 and 0.5 mm, Qingdao Haiyang Chemical Co., Ltd., Qingdao, China). Vanillin-sulfuric acid was used as the spray reagent for TLC. Analytical and semi-preparative high-performance liquid chromatography (HPLC and semi-preparative HPLC) was performed using the C_18_ column (4.6 × 250 and 9.4 × 250 mm, 5 μm, Agilent) on an Agilent 1260 series system (Agilent, Palo Alto, CA, USA). Morphology of root tip cells was observed by Leica GALEN-III optical microscope (Nanjing Jiangnan Photoelectric (Group) Co., Ltd., Nanjing, China) and SU8020 scanning electron microscope (Hitachi, Tokyo, Japan). Relative conductivity was measured using DDSJ-308F conductivity meter (INESA Scientific Instrument Co., Ltd., Shanghai, China). The ethylene content was tested using GC-2010Pro gas chromatograph (Shimadzu, Kyoto, Japan). All chemical agents were purchased from Sinopharm Chemical Reagent company (Shanghai, China).

### 4.2. Fungal Material

The phytopathogenic fungus *B. cookei* SYBL03 was provided from Institute of Plant Protection, Liaoning Academy of Agricultural Sciences, China. The strain was isolated from the diseased leaves of target leaf spot of sorghum, a crop growing in Shenyang, Liaoning province, China. On the basis of the colony and spore morphology as well as the sequence analysis of the internal transcribed spacer (ITS) of nuclear ribosomal DNA, the strain SYBL03 was identified as *B. cookei* [69]. Working stocks were prepared on the slants containing potato dextrose agar (PDA) medium [70] and stored at 4 °C.

### 4.3. Fermentation and Extraction

*B. cookei* SYBL03 culture was initiated by transferring pieces of PDA medium containing mycelium to a fresh PDA slant. A loop full of the well-grown culture was transferred to a 250 mL Erlenmeyer flask with 100 mL modified PD (PDm) medium containing infusion from 20 g potatoes, dextrose 2 g, peptone 0.1 g, K_2_HPO_4_ 0.1 g, MgSO_4_ 0.05 g, Vitamin B_1_ 1 mg in distilled water at pH 7.0. The flasks were incubated in a rotary incubator shaker at 28 °C and 180 rpm for 48 h and were used as seed culture for subsequent experiments. Modified potato sugar (PSm) medium (infusion from 200 g potatoes, sucrose 20 g, NaNO_3_ 1 g, KCl 0.5 g, MgSO₄·7H₂O 0.5 g, FeSO_4_·7H₂O 0.02 g, CuSO_4_·5H₂O 0.02 g and distilled water up to 1 L, natural pH value) was used for fermentation production. The seed cultures (14 mL) were inoculated in 500 mL flasks containing 200 mL of PSm medium each. The flasks were incubated in rotary shakers at 28 °C and 180 rpm for 7 d. A total of 150 L fermentation culture was obtained. The broth and mycelia were collected by centrifugation at 9000 rpm for 20 min, respectively.

Mycelia were dried, crushed into powder, and extracted five times in 3 volumes of 80% ice-cold acetone. The organic solution was collected by filtration, and the combined filtrates were concentrated to remove acetone. The aqueous residue was repeatedly extracted with an equal volume of EtOAc. The organic layer was concentrated under reduced pressure to produce 9.7 g of the mycelium extract.

Twelve grams of the Amberlite XAD-16N resin was added to each 500 mL Erlenmeyer flask with 300 mL of the cell-free supernatant. The resin was collected by filtration after shaking at 20 °C and 120 rpm for 12 h, dried in an oven at 25 °C, and desorbed five times with methanol (MeOH). Desorbed solution was pooled and concentrated by rotary evaporator under vacuum to yield the total residue, which was then suspended in water and repeatedly extracted with EtOAc and then concentrated, as described above. Finally, the broth extract (27 g) was obtained.

### 4.4. Fractionation and Purification of Compounds

The EtOAc extract of mycelia was fractionated by column chromatography (CC) over silica gel using an increasing gradient of EtOAc-MeOH (99:1, 95:5, 90:10, 80:20, 50:50, 0:100, *v*/*v*) to afford six main fractions (A–F). Fraction B (298 mg) was purified by CC over Sephadex LH-20 eluted with CH_2_Cl_2_-MeOH (1:1, *v*/*v*) to yield two subfractions (B1 and B2). Subfraction B1 (180 mg) was dissolved in CH_2_Cl_2_-MeOH (1:1, *v*/*v*). The insoluble part (61 mg) was isolated by semi-preparative HPLC (MeOH-H_2_O, 93:7, *v*/*v*) to yield compounds **1** (8.2 mg, *t*_R_ = 23.34 min) and **2** (32 mg, *t*_R_ = 31.21 min). Fraction C (105 mg) was dissolved in methanol. The insoluble part (52 mg) was recrystallized from MeOH and further purified by Sephadex LH-20 column eluted with CH_2_Cl_2_-MeOH (1:1, *v*/*v*) to give compound **3** (35 mg). Fraction D (158 mg) was repeatedly purified by Sephadex LH-20 column eluted with CH_2_Cl_2_-MeOH (1:1, *v*/*v*) and was recrystallized from MeOH to obtain compound **4** (11 mg). Fraction E (75 mg) was subjected to CC over Sephadex LH-20 eluted with CH_2_Cl_2_-MeOH (1:1, *v*/*v*) to yield subfractions E1 and E2. As the solvent evaporated, some components precipitated out of the eluting solvent of subfraction E1. The precipitate (30 mg) was further purified by semi-preparative HPLC (100% MeOH) to afford compounds **5** (8.2 mg, *t*_R_ = 16.10 min) and **6** (12 mg, *t*_R_ = 18.88 min).

The EtOAc extract of the cell-free supernatant was subjected to silica gel CC and eluted with a gradient of PE-EtOAc (9:1, 8:2, 7:3, 5:5, 4:6, 2:8, 0:10, *v*/*v*) to obtain five fractions (A′–E′). Fraction C′ (2.78 g) was purified by CC over silica gel eluted with a step-wise gradient of PE-EtOAc (7:3 to 4:6, *v*/*v*) to yield compound **3** (19 mg) and mixed subfraction C1′ (1.25 g). Subfraction C1′ was further separated through Sephadex LH-20 column eluted with CH_2_Cl_2_-MeOH (1:1, *v*/*v*) and PTLC (PE-CH_2_Cl_2_-EtOH, 3:8:2, *v*/*v*), to afford compound **7** (752 mg). Fraction D′ (1.75 g) was applied to CC over silica gel eluted with PE-EtOAc (2:8, *v*/*v*) to give subfractions D1′ and D2′. Subfraction D1′ (125 mg) was repeatedly purified via Sephadex LH-20 column with CH_2_Cl_2_-MeOH (1:1, *v*/*v*) and semi-preparative HPLC (MeOH-H_2_O, 7:3, *v*/*v*) to afford compound **8** (23 mg, *t*_R_ = 29.56 min).

#### 4.4.1. 5α,8α-Epidioxyergosta-6,9(11),22-trien-3β-ol (**1**)

White powder; ^1^H-NMR (600 MHz, CD_3_OD): *δ*_H_ 6.65 (1H, d, *J* = 8.6 Hz, H-7), 6.30 (1H, d, *J* = 8.6 Hz, H-6), 5.48 (1H, dd, *J* = 6.1, 1.9 Hz, H-11), 5.26 (1H, dd, *J* = 15.3, 7.7 Hz, H-23), 5.20 (1H, dd, *J* = 15.3, 8.3 Hz, H-22), 3.80 (1H, m, H-3), 1.10 (3H, s, H-19), 1.02 (3H, d, *J* = 6.6 Hz, H-21), 0.93 (3H, d, *J* = 6.8 Hz, H-28), 0.86 (3H, d, *J* = 6.8 Hz, H-26), 0.84 (3H, d, *J* = 6.8 Hz, H-27), 0.77 (3H, s, H-18); ^13^C-NMR (150 MHz, CD_3_OD): *δ*_C_ 144.2 (s, C-9), 136.9 (d, C-6), 136.7 (d, C-22), 133.6 (d, C-23), 131.8 (d, C-7), 120.7 (d, C-11), 84.0 (s, C-5), 79.7 (s, C-8), 66.8 (d, C-3), 57.2 (d, C-17), 49.6 (d, C-14), 44.8 (s, C-13), 44.3 (d, C-24), 42.4 (t, C-12), 41.3 (d, C-20), 39.2 (s, C-10), 36.9 (t, C-4), 34.4 (d, C-25), 33.7 (t, C-1), 31.3 (t, C-2), 29.8 (t, C-16), 25.9 (q, C-19), 21.8 (q, C-21), 21.2 (t, C-15), 20.4 (q, C-26), 20.1 (q, C-27), 18.2 (q, C-28), 13.4 (q, C-18); ESI-MS *m*/*z* 449.31 [M + Na]^+^ (calcd. for C_28_H_42_O_3_Na^+^, 449.3032).

#### 4.4.2. Ergosterol Peroxide (**2**)

Colorless needle crystals; ^1^H-NMR (600 MHz, CD_3_OD): *δ*_H_ 6.53 (1H, d, *J* = 8.5 Hz, H-6), 6.25 (1H, d, *J* = 8.5 Hz, H-7), 5.24 (1H, dd, *J* = 15.2, 7.7 Hz, H-23), 5.18 (1H, dd, *J* = 15.2, 8.3 Hz, H-22), 3.77 (1H, m, H-3), 1.01 (3H, d, *J* = 6.6 Hz, H-21), 0.93 (3H, d, *J* = 6.8 Hz, H-28), 0.90 (3H, s, H-19), 0.85 (3H, s, H-18), 0.85 (3H, d, *J* = 6.8 Hz, H-26), 0.83 (3H, d, *J* = 6.8 Hz, H-27); ^13^C-NMR (150 MHz, CD_3_OD): *δ*_C_ 136.8 (d, C-6, C-22), 133.5 (d, C-23), 131.7 (d, C-7), 83.5 (s, C-5), 80.7 (s, C-8), 67.0 (d, C-3), 57.6 (d, C-17), 53.1 (d, C-14), 52.7 (d, C-9), 45.8 (s, C-13), 44.3 (d, C-24), 41.1 (d, C-20), 40.7 (t, C-12), 38.2 (s, C-10), 37.8 (t, C-4), 35.9 (t, C-1), 34.4 (d, C-25), 30.9 (t, C-2), 29.8 (t, C-16), 24.4 (t, C-11), 21.6 (q, C-21), 21.4 (t, C-15), 20.5 (q, C-26), 20.1 (q, C-27), 18.6 (q, C-19), 18.2 (q, C-28), 13.3 (q, C-18): ESI-MS *m*/*z* 429.31 [M + H]^+^ (calcd. for C_28_H_45_O_3_^+^, 429.3369).

#### 4.4.3. (–)-Ditryptophenaline (**3**)

White crystal; ^1^H-NMR (600 MHz, CD_3_OD): *δ*_H_ 7.57 (4H, dd, *J* = 7.6, 7.4 Hz, H-20, H-22, H-20′, H-22′), 7.50 (2H, t, *J* = 7.4 Hz, H-21, H-21′), 7.15 (4H, d, *J* = 7.6 Hz, H-19, H-23, H-19′, H-23′), 7.04 (2H, dd, *J* = 7.9, 7.4 Hz, H-7, H-7′), 6.95 (2H, d, *J* = 7.5 Hz, H-5, H-5′), 6.65 (2H, dd, *J* = 7.5, 7.4 Hz, H-6, H-6′), 6.57 (2H, d, *J* = 7.9 Hz, H-8, H-8′), 5.00 (2H, s, H-2, H-2′), 4.40 (2H, m, H-15, H-15′), 3.63 (2H, br dd, *J* = 12.0, 4.8 Hz, H-11, H-11′), 3.49 (2H, dd, *J* = 14.3, 2.8 Hz, H-17a, H-17a′), 3.30 (2H, overlapped, H-17b, H-17b′), 3.03 (6H, s, H-24, H-24′), 1.88 (2H, dd, *J* = 12.1, 4.8 Hz, H-12a, H-12a′), 1.46 (2H, dd, *J* = 12.1, 12.0 Hz, H-12b, H-12b′);^13^C-NMR (150 MHz, CD_3_OD): *δ*_C_ 167.6 (s, C-13, C-13′), 165.5 (s, C-16, C-16′), 152.7 (s, C-9, C-9′), 136.1 (s, C-18, C-18′), 130.7 (d, C-7, C-7′), 130.5 (d, C-19, C-23, C-19′, C-23′), 130.4 (d, C-20, C-22, C-20′, C-22′), 129.2 (d, C-21, C-21′), 127.7 (s, C-4, C-4′), 126.4 (d, C-5, C-5′), 119.1 (d, C-6, C-6′), 110.2 (d, C-8, C-8′), 79.9 (d, C-2, C-2′), 64.4 (d, C-15, C-15′), 60.4 (s, C-3, C-3′), 59.6 (d, C-11, C-11′), 37.4 (t, C-12, C-12′), 36.9 (t, C-17, C-17′), 33.1 (q, C-24, C-24′); ESI-MS *m*/*z* 693.34 [M + H]^+^ (calcd. for C_42_H_41_N_6_O_4_^+^, 693.3189).

#### 4.4.4. 1,3,9-Trimethyluric Acid (**4**)

White powder; ^1^H-NMR (600 MHz, CD_3_OD): *δ*_H_ 3.73 (3H, s, H-11), 3.60 (3H, s, H-12), 3.32 (3H, s, H-10); ^13^C-NMR (150 MHz, CD_3_OD): *δ*_C_ 154.7 (s, C-6), 154.0 (s, C-8), 152.5 (s, C-2), 138.5 (s, C-4), 99.6 (s, C-5), 31.9 (q, C-11), 30.4 (q, C-12), 28.6 (q, C-10); ESI-MS *m*/*z* 211.05 [M + H]^+^ (calcd. for C_8_H_11_N_4_O_3_^+^, 211.0831).

#### 4.4.5. Cerebroside A (**5**)

White powder; ^1^H-NMR (600 MHz, CD_3_OD): *δ*_H_ 7.71 (1H, d, *J* = 9.4 Hz, NH), 5.83 (1H, dt, *J* = 15.3, 6.6 Hz, H-4′), 5.71 (1H, dt, *J* = 15.4, 6.3 Hz, H-5), 5.49 (1H, dd, *J* = 15.3, 6.0 Hz, H-3′), 5.45 (1H, dd, *J* = 15.4, 7.4 Hz, H-4), 5.14 (1H, t, *J* = 6.6 Hz, H-8), 4.43 (1H, d, *J* = 6.0 Hz, H-2′), 4.27 (1H, d, *J* = 7.8 Hz, H-1′’), 4.13 (1H, dd, *J* = 10.4, 5.6 Hz, H-1a), 4.12 (1H, m, H-3), 3.97 (1H, m, H-2), 3.86 (1H, br d, *J* = 11.9 Hz, H-6′’a), 3.70 (1H, dd, *J* = 10.4, 3.3 Hz, H-1b), 3.66 (1H, dd, *J* = 11.9, 4.6 Hz, H-6″b), 3.17–3.37 (4H, m, H-2″, H-3″, H-4″, H-5″), 2.00–2.09 (6H, m, H-6, H-7, H-5′), 1.97 (2H, t, *J* = 7.5 Hz, H-10), 1.59 (3H, br s, H-19), 1.21–1.42 (34H, m, H-11–H-17, H-6′–H-15′), 0.89 (6H, t, *J* = 6.8 Hz, H-18, H-16′); ^13^C-NMR (150 MHz, CD_3_OD): *δ*_C_ 175.4 (s, C-1′), 136.7 (s, C-9), 134.7 (d, C-5), 134.5 (d, C-4′), 131.0 (d, C-4), 129.0 (d, C-3′), 124.9 (d, C-8), 104.7 (d, C-1″), 78.0 (d, C-5″), 77.9 (d, C-3″), 75.0 (d, C-2″), 74.1 (d, C-2′), 72.9 (d, C-3), 71.6 (d, C-4″), 69.7 (t, C-1), 62.7 (t, C-6″), 54.6 (d, C-2), 40.8 (t, C-10), 33.8 (t, C-6), 33.4 (t, C-5′), 33.1 (t, C-16, C-14′), 30.2–30.8 (t, C-12–C-15, C-6′–C-13′), 29.1 (t, C-11), 28.8 (t, C-7), 23.8 (t, C-17, C-15′), 16.2 (q, C-19), 14.5 (q, C-18, C-16′); ESI-MS *m*/*z* 726.52 [M + H]^+^ (calcd. for C_41_H_76_NO_9_^+^, 726.5520).

#### 4.4.6. Cerebroside B (**6**)

White powder; ^1^H-NMR (600 MHz, CD_3_OD): *δ*_H_ 7.71 (1H, d, *J* = 9.4 Hz, NH), 5.73 (1H, dt, *J* = 15.3, 6.3 Hz, H-5), 5.48 (1H, dd, *J* = 15.3, 7.4 Hz, H-4), 5.14 (1H, t, *J* = 6.5 Hz, H-8), 4.26 (1H, d, *J* = 7.8 Hz, H-1″), 4.13 (1H, m, H-3), 4.11 (1H, dd, *J* = 10.3, 5.6 Hz, H-1a), 3.98 (2H, m, H-2, H-2′), 3.86 (1H, br d, *J* = 11.8 Hz, H-6″a), 3.70 (1H, dd, *J* = 10.3, 3.3 Hz, H-1b), 3.66 (1H, dd, *J* = 11.8, 4.4 Hz, H-6″b), 3.16–3.37 (4H, m, H-2″, H-3″, H-4″, H-5″), 2.06 (4H, m, H-6, H-7), 1.97 (2H, t, *J* = 7.5 Hz, H-10), 1.70 (1H, m, H-3′a), 1.59 (3H, br s, H-19), 1.55 (1H, m, H-3′b), 1.20–1.44 (38H, m, H-11–H-17, H-4′–H-15′), 0.89 (6H, t, *J* = 6.9 Hz, H-18, H-16′); ^13^C-NMR (150 MHz, CD_3_OD): *δ*_C_ 177.2 (s, C-1′), 136.8 (s, C-9), 134.7 (d, C-5), 131.1 (d, C-4), 124.8 (d, C-8), 104.7 (d, C-1″), 78.0 (d, C-5″), 77.9 (d, C-3″), 75.0 (d, C-2″), 73.1 (d, C-2′), 72.9 (d, C-3), 71.6 (d, C-4″), 69.7 (t, C-1), 62.7 (t, C-6″), 54.6 (d, C-2), 40.8 (t, C-10), 35.9 (t, C-3′), 33.8 (t, C-6), 33.1 (t, C-16, C-14′), 30.4–30.8 (t, C-12–C-15, C-5′–C-13′), 29.1 (t, C-11), 28.7 (t, C-7), 26.2 (t, C-4′), 23.8 (t, C-17, C-15′), 16.1 (q, C-19), 14.5 (q, C-18, C-16′); ESI-MS *m*/*z* 728.56 [M + H]^+^ (calcd. for C_41_H_78_NO_9_^+^, 728.5677).

#### 4.4.7. Cyclo-*N*-methylphenylalanyltryptophenyl (**7**)

White powder; ^1^H-NMR (600 MHz, CD_3_OD): *δ*_H_ 7.52 (1H, d, *J* = 7.9 Hz, H-6), 7.34 (1H, d, *J* = 8.1 Hz, H-3), 7.23 (2H, dd, *J* = 7.5, 7.3 Hz, H-21, H-23), 7.18 (1H, t, *J* = 7.3 Hz, H-22), 7.12 (1H, dd, *J* = 8.1, 7.1 Hz, H-4), 7.04 (1H, dd, *J* = 7.9, 7.1 Hz, H-5), 6.95 (1H, s, H-9), 6.79 (2H, d, *J* = 7.5 Hz, H-20, H-24), 4.11 (1H, dd, *J* = 7.2, 3.9 Hz, H-11), 4.04 (1H, dd, *J* = 6.3, 4.3 Hz, H-15), 2.94 (1H, dd, *J* = 14.5, 3.9 Hz, H-10a), 2.72 (1H, dd, *J* = 14.2, 4.3 Hz, H-18a), 2.68 (3H, s, H-14), 2.19 (1H, dd, *J* = 14.5, 7.2 Hz, H-10b), 2.03 (1H, dd, *J* = 14.2, 6.3 Hz, H-18b); ^13^C-NMR (150 MHz, CD_3_OD): *δ*_C_ 168.9 (s, C-16), 168.4 (s, C-12), 138.1 (s, C-2), 138.0 (s, C-19), 130.8 (d, C-20, C-24), 129.7 (d, C-21, C-23), 128.7 (s, C-7), 128.2 (d, C-22), 125.6 (d, C-9), 122.7 (d, C-4), 120.1 (d, C-5), 119.7 (d, C-6), 112.6 (d, C-3), 109.9 (s, C-8), 64.9 (d, C-15), 57.2 (d, C-11), 39.4 (t, C-18), 34.3 (q, C-14), 31.7 (t, C-10); ESI-MS *m*/*z* 348.17 [M + H]^+^ (calcd. for C_21_H_22_N_3_O_2_^+^, 348.1712).

#### 4.4.8. (2S)-3,3-di-1*H*-indol-3-yl-1,2-propanediol (**8**)

White powder; [α]_D_^20^ +39.4° (c 0.29, MeOH); ^1^H-NMR (600 MHz, CDCl_3_): *δ*_H_ 7.98, 8.06 (each 1H, br s, NH-1′, NH-1″), 7.62, 7.67 (each 1H, d, *J* = 8.0 Hz, H-4′, H-4″), 7.31, 7.33 (each 1H, d, *J* = 8.2 Hz, H-7′, H-7″), 7.15–7.20 (2H, m, H-6′, H-6″), 7.09, 7.19 (each 1H, br s, H-2′, H-2″), 7.05–7.11 (2H, m, H-5′, H-5″), 4.73 (1H, d, *J* = 7.1 Hz, H-1), 4.53 (1H, ddd, *J* = 7.1, 6.8, 3.4 Hz, H-2), 3.75 (1H, dd, *J* = 11.3, 3.4 Hz, H-3a), 3.63 (1H, dd, *J* = 11.3, 6.8 Hz, H-3b); ^13^C-NMR (150 MHz, CDCl_3_): *δ*_C_ 136.3, 136.4 (s, C-7a′, C-7a″), 126.8, 127.4 (s, C-3a′, C-3a″), 122.3, 122.8 (d, C-2′, C-2″), 122.1, 122.3 (d, C-6′, C-6″), 119.5, 119.7 (d, C-5′, C-5″), 119.2, 119.4 (d, C-4′, C-4″), 115.3, 116.8 (s, C-3′, C-3″), 111.2 (d, C-7′, C-7″), 74.8 (d, C-2), 65.3 (t, C-3), 36.9 (d, C-1); ESI-MS *m*/*z* 305.11 [M – H]^–^ (calcd. for C_19_H_17_N_2_O_2_^−^, 305.1290).

### 4.5. Herbicidal Activity Assay

The compounds to be tested were dissolved in dimethyl sulfoxide (DMSO) at a concentration of 80 mg/mL, and solutions were diluted with 0.1% Tween 80 to make a stock solution concentration of 800 µg/mL. The required dilutions were subsequently made from the stock solution by adding 1% DMSO water with 0.1% Tween 80. The solution containing the same concentration of DMSO and Tween 80 was used as the control treatment.

Mature weed seeds of six species (*E. crus-galli*, *S. viridis*, *D. sanguinalis*, *A. retroflexus*, *S. nigrum* and *C. bursa-pastoris*) were collected from the suburban roadsides, hillsides, and wetlands of Liaoning Province, China. Seeds were kept at 4 °C until further use. Weed seeds were surface sterilized by treatment with 0.2% sodium hypochlorite solution for 10 min, followed by rinsing in sterile distilled water at least three times. Except for *C. bursa-pastoris* (40 seeds), 30 seeds were equidistantly placed in a Petri dish (diameter = 90 mm) containing double-layer filter paper. Afterward, each Petri dish was moistened with the solution to be tested (5 mL) and kept at 26 ± 1 °C, with 85% humidity and a 12 h photoperiod. At least three replicates were taken for each treatment of each compound. Seeds were considered to have germinated if the root extended at least 2 mm long. The number of germinated seeds was recorded continuously for seven days. Except for *C. bursa-pastoris* (144 h), the root and shoot lengths of germinated seeds were measured after 96 h. Germination rate was calculated as the total number of germinated seeds at the end of experiment out of the total number of seeds tested. Inhibition rate is calculated by using Formula (1).

Inhibition (%) = (*C* − *T*)/*C* × 100 (1)

where *C* represents parameters of the control group and *T* represents parameters of the treatment group.

### 4.6. Light Microscopy

After treatment with compound **7** for 96 h at concentration of 400 μg/mL, the roots of *A. retroflexus* seedlings were kept in distilled water to observe the elongation-zone cells and root hairs, and then photographed under a Leica GALEN-III optical microscope (Nanjing Jiangnan Photoelectric (Group) Co., Ltd., Nanjing, China). Then, 1% DMSO water with 0.1% Tween 80 was used as the control treatment.

### 4.7. Scanning Electron Microscopy (SEM)

In order to clarify the herbicidal mechanism of compound **7**, the cellular morphological changes of *A. retroflexus* roots were observed using SEM. After treatment with or without compound **7** (400 μg/mL) for 96 h, the roots were fixed in 2.5% glutaraldehyde solution for 24 h at 4 °C, dehydrated with an ethanol series (50%, 70% and 100%), dried by critical point dryer (Tousimis Autosamdri-815, Series, Tousimis, Rockville, MD, USA) for 1 h. The sputter coater (HIYACHI MC1000, Hitachi, Tokyo, Japan) was used to deposit a thin gold coating. The samples were observed under SEM (Hitachi SU8020, Hitachi, Tokyo, Japan) at 5 kV.

### 4.8. Relative Conductivity Assay

Seedlings grown for 96 h in the presence or absence of compound **7** (400 μg/mL) were used to assay electrolyte leakage. Fresh root samples (150 mg) were suspended in a test tube containing 10 mL of deionized water and incubated at 25 °C for 12 h to measure the conductivity of the solution (*R*_1_). The test tubes were then heated into a thermostatic water bath at 100 °C for 15 min to completely kill the tissues, and the conductivity value of the same sample was measured again after being cooled down (*R*_2_). The experiment was run in triplicates. The relative conductivity (REC) was calculated using Formula (2).

REC (%) = *R*_1_/*R*_2_ × 100 (2)

### 4.9. Ethylene Content Assay

Seedlings of *A. retroflexus* were incubated for 96 h in the presence or absence of compound **7** (400 μg/mL). The root samples (100 mg) were sonicated for 30 min in a headspace bottle containing 0.1 g of NaCl, and 1 mL of 10 mol/L NaOH solution. After 24 h, ethylene content was determined by gas chromatography (GC) as described by Liu et al. [71].

### 4.10. Statistical Analyses

The measurement data were expressed as mean ± standard error of at least three biological replicates. One-way analysis of variance (ANOVA) with Holm–Sidak method or the two-tailed *t*-test was performed using SigmaPlot 12.5. Differences were considered significant at (*) *p* < 0.05, (**) *p* < 0.01 and (***) *p* < 0.001.

## Figures and Tables

**Table 1:** Effects of compounds **1**–**8** at 200 μg/mL on the seedling growth of *A. retroflexus* and *E. crus-galli*.

Compounds	*A. Retroflexus*		*E. crus-galli*	
	Shoot Length (mm)	Root Length (mm)	Shoot Length (mm)	Root Length (mm)
**1**	11.0 ± 0.3 ^b^	20.3 ± 0.3 ^b^	30.2 ± 2.1 ^b^	9.6 ± 0.3 ^b^
**2**	8.7 ± 0.3 ^c^	11.1 ± 0.3 ^c^	33.3 ± 2.5 ^b^	9.1 ± 0.7 ^b^
**3**	15.0 ± 0.6 ^a^	24.0 ± 0.4 ^a^	31.9 ± 1.6 ^b^	9.6 ± 0.9 ^b^
**4**	14.4 ± 0.4 ^a^	24.8 ± 0.5 ^a^	32.8 ± 2.2 ^b^	9.8 ± 1.1 ^b^
**5**	16.1 ± 0.3 ^a^	25.4 ± 0.2 ^a^	31.4 ± 2.1 ^b^	9.8 ± 1.6 ^b^
**6**	15.7 ± 0.4 ^a^	26.9 ± 0.3 ^a^	31.0 ± 2.9 ^b^	10.2 ± 1.0 ^b^
**7**	7.7 ± 0.3 ^c^	12.2 ± 0.3 ^c^	46.0 ± 2.7 ^a^	22.8 ± 1.7 ^a^
**8**	14.8 ± 0.5 ^a^	23.9 ± 0.3 ^a^	32.3 ± 2.4 ^b^	10.1 ± 1.6 ^b^
Control	14.3 ± 0.4 ^a^	25.2 ± 0.7 ^a^	31.4 ± 2.7 ^b^	9.7 ± 0.7 ^b^

Data shown are the mean of three independent experiments and presented as mean ± SE. Means followed by the different letters in the same column indicate significant differences (*p* < 0.05) among groups using one-way analysis of variance (ANOVA).

## Data Availability

The original contributions presented in the study are included in the article/Supplementary Materials, further inquiries can be directed to the corresponding author.

## Supplementary Materials

The following supporting information can be downloaded at: https://www.mdpi.com/article/10.3390/molecules29133040/s1, Figures S1–S25: The 1D NMR spectra of compounds **1**–**8**, 2D NMR spectra of **4**.
