# Supplementary material for: A Phytotoxin with Selective Herbicidal Activity and Related Metabolites from the Phytopathogenic Fungus Bipolaris cookei SYBL03
Source: Molecules. 2024 Jun 26;29(13):3040. doi: 10.3390/molecules29133040 (PMC11243656; doi:10.3390/molecules29133040)
Supplement: Supplementary file 1 [file molecules-29-03040-s001.zip › molecules-3046078-supplementary.pdf]

Supplementary Materials

A Phytotoxin with Selective Herbicidal Activity and Related Metabolites from the Phytopathogenic Fungus *Bipolaris cookei* SYBL03

Haiyan Li [1,2], Jingzhuo Hou [2], Bing Li [2], Lizhong Zhang [3] and Zhiguo Yu [1,*]

1 College of Plant Protection, Shenyang Agricultural University, Shenyang 110866, People's Republic of China; lihaiyan98@syuct.edu.cn

2 College of Environmental and Safety Engineering, Shenyang University of Chemical Technology, Shenyang 110142, People's Republic of China; z2023538@stu.syuct.edu.cn (J.H.); libinglucky@163.com (B.L.)

3 College of Computer Science and Technology, Shenyang University of Chemical Technology, Shenyang 110142, People's Republic of China; zhanglz_2009@syuct.edu.cn

* Correspondence: zyu@syau.edu.cn; Tel.: +86-24-88487148 (Z.Y.)

Contents

Figure S1. The ^{1}H-NMR spectrum of 9,11-dehydroergosterol peroxide (**1**) in CD_3OD....1

Figure S2. The ^{13}C-NMR spectrum of 9,11-dehydroergosterol peroxide (**1**) in CD_3OD...1

Figure S3. The ESI-MS spectrum of 9,11-dehydroergosterol peroxide (**1**) 2

Figure S4. The ^{1}H-NMR spectrum of ergosterol peroxide (**2**) in CD_3OD 2

Figure S5. The ^{13}C-NMR spectrum of ergosterol peroxide (**2**) in CD_3OD 3

Figure S6. The ESI-MS spectrum of ergosterol peroxide (**2**) 3

Figure S7. The ^{1}H-NMR spectrum of (–)-ditryptophenaline (**3**) in CD_3OD. 4

Figure S8. The ^{13}C-NMR spectrum of (–)-ditryptophenaline (**3**) in CD_3OD....................4

Figure S9. The ESI-MS spectrum of (–)-ditryptophenaline (**3**) 5

Figure S10. The ^{1}H-NMR spectrum of 1,3,9-trimethyluric acid (**4**) in CD_3OD 5

Figure S11. The ^{13}C-NMR spectrum of 1,3,9-trimethyluric acid (**4**) in CD_3OD 6

Figure S12. The HMBC spectrum of 1,3,9-trimethyluric acid (**4**) in CD_3OD 6

Figure S13. The ESI-MS spectrum of 1,3,9-trimethyluric acid (**4**)...................................7

Figure S14. The ^{1}H-NMR spectrum of cerebroside A (**5**) in CD_3OD...............................7

Figure S15. The ^{13}C-NMR spectrum of cerebroside A (**5**) in CD_3OD.............................8

Figure S16. The ESI-MS spectrum of cerebroside A (**5**) 8

Figure S17. The ^{1}H-NMR spectrum of cerebroside B (**6**) in CD_3OD 9

Figure S18. The ^{13}C-NMR spectrum of cerebroside B (**6**) in CD_3OD 9

Figure S19. The ESI-MS spectrum of cerebroside B (**6**) 10

Figure S20. The ^{1}H-NMR spectrum of *cyclo*-*N*-methylphenylalanyltryptophenyl (**7**) in CD_3OD 10

Figure S21. The ^{13}C-NMR spectrum of *cyclo*-*N*-methylphenylalanyltryptophenyl (**7**) in

CD_3OD 11

Figure S22. The ESI-MS of *cyclo*-*N*-methylphenylalanyltryptophenyl (**7**) 11

Figure S23. The ^{1}H-NMR spectrum of (2*S*)-3,3-di-1*H*-indol-3-yl-1,2-propanediol (**8**) in $CDCl_3$ 12

Figure S24. The ^{13}C-NMR spectrum of (2*S*)-3,3-di-1*H*-indol-3-yl-1,2-propanediol (**8**) in $CDCl_3$ 12

Figure S25. The ESI-MS spectrum of (2*S*)-3,3-di-1*H*-indol-3-yl-1,2-propanediol (**8**).....13

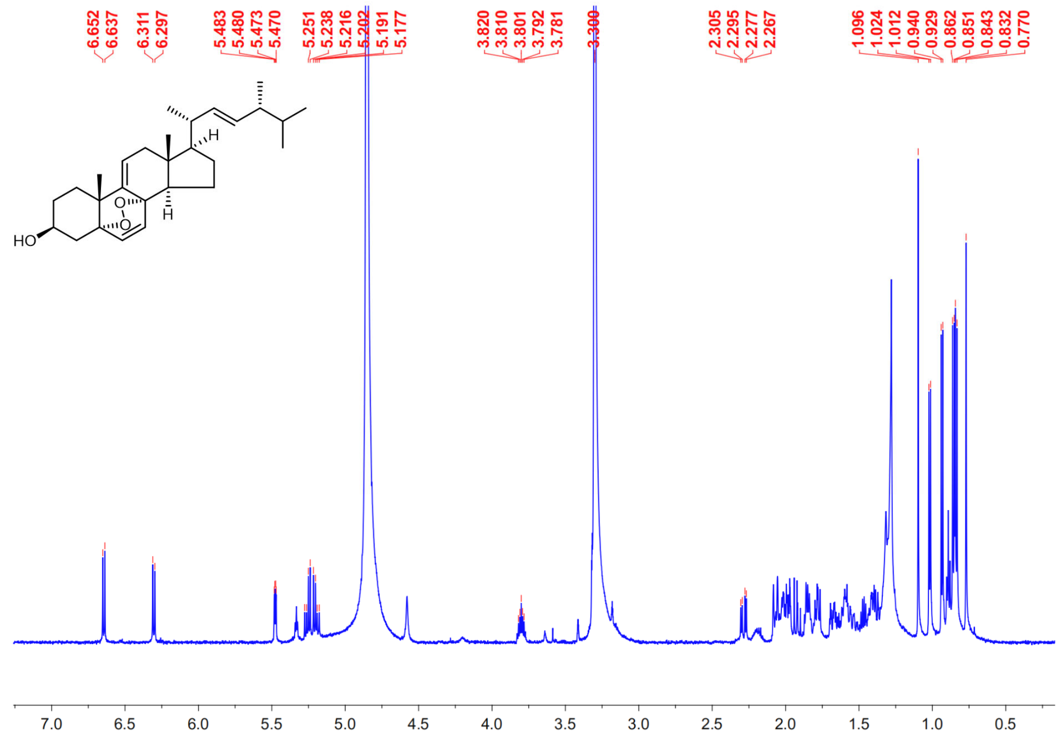

Figure S1. The ^{1}H-NMR spectrum of 9,11-dehydroergosterol peroxide (**1**) in CD_3OD

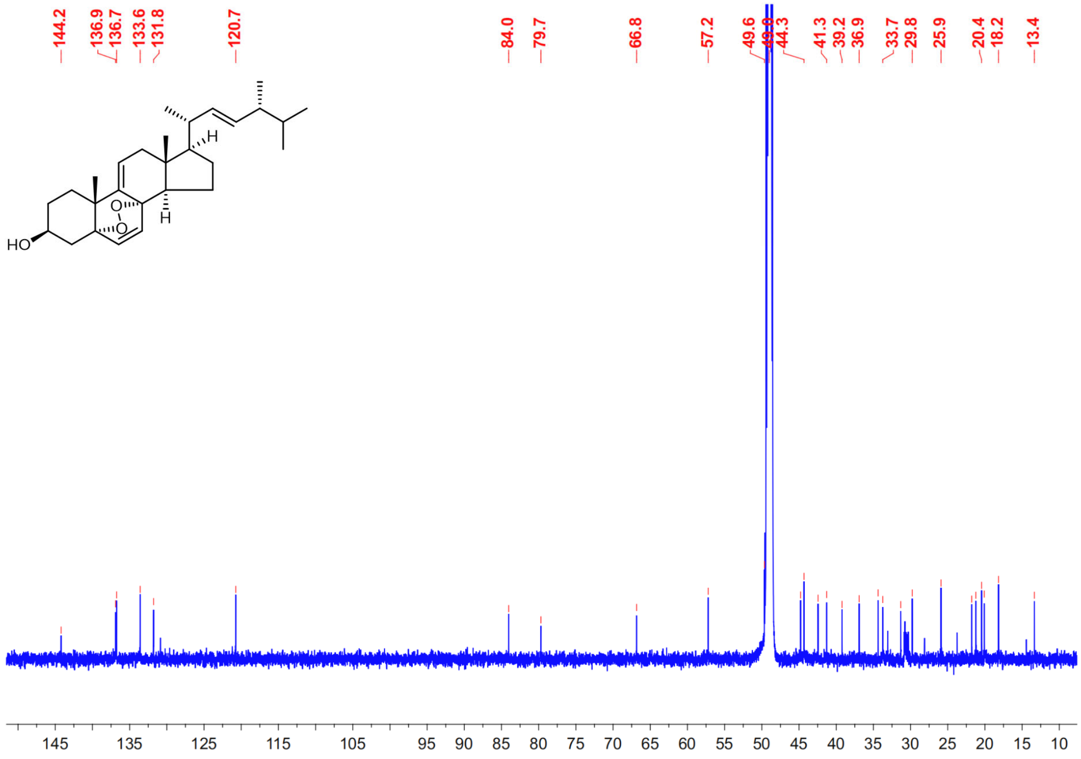

Figure S2. The ^{13}C-NMR spectrum of 9,11-dehydroergosterol peroxide (**1**) in CD_3OD

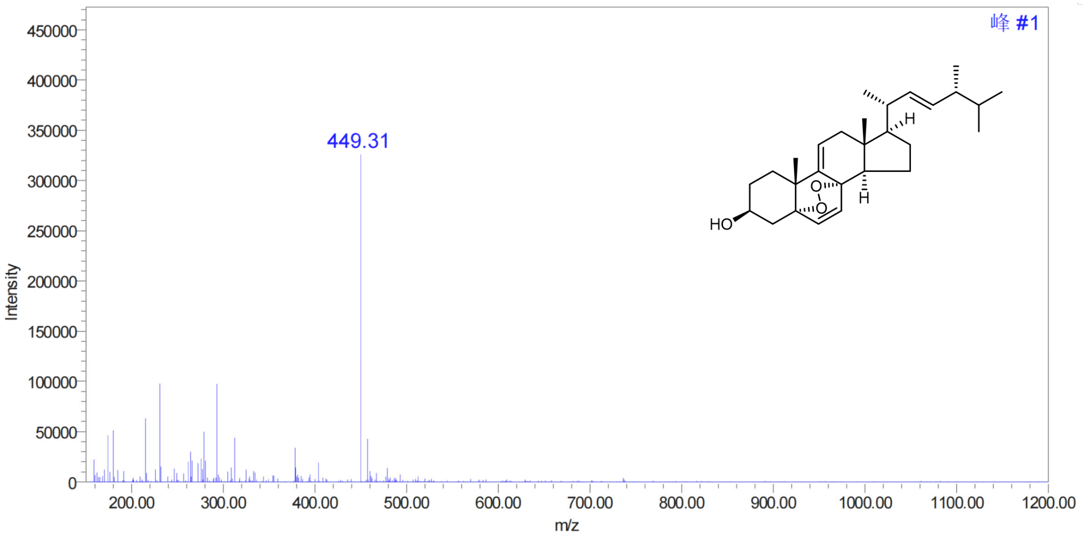

Figure S3. The ESI-MS spectrum of 9,11-dehydroergosterol peroxide (**1**)

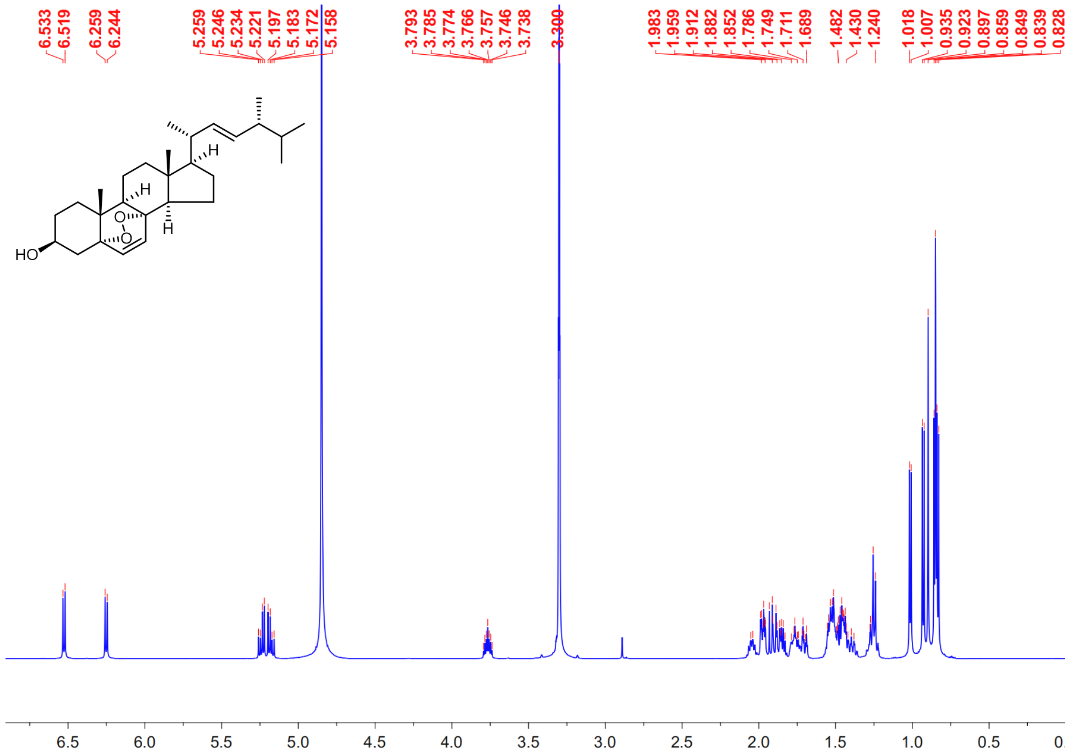

Figure S4. The ^{1}H-NMR spectrum of ergosterol peroxide (**2**) in CD_3OD

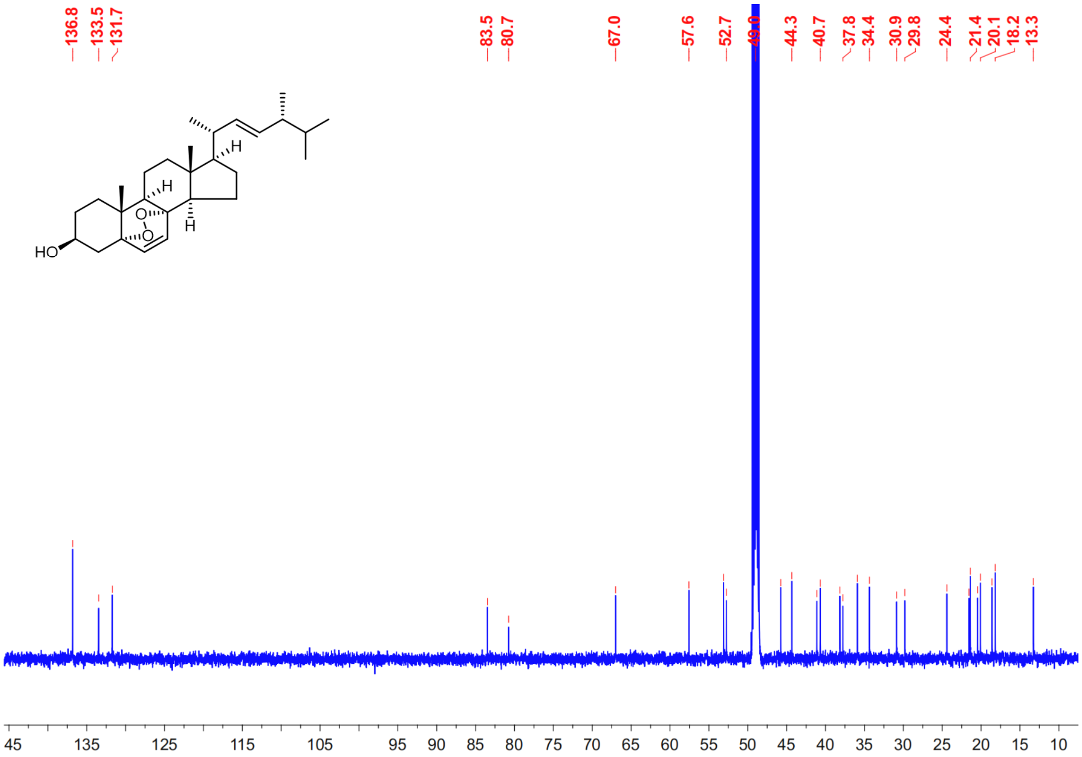

Figure S5. The ^{13}C-NMR spectrum of ergosterol peroxide (**2**) in CD_3OD

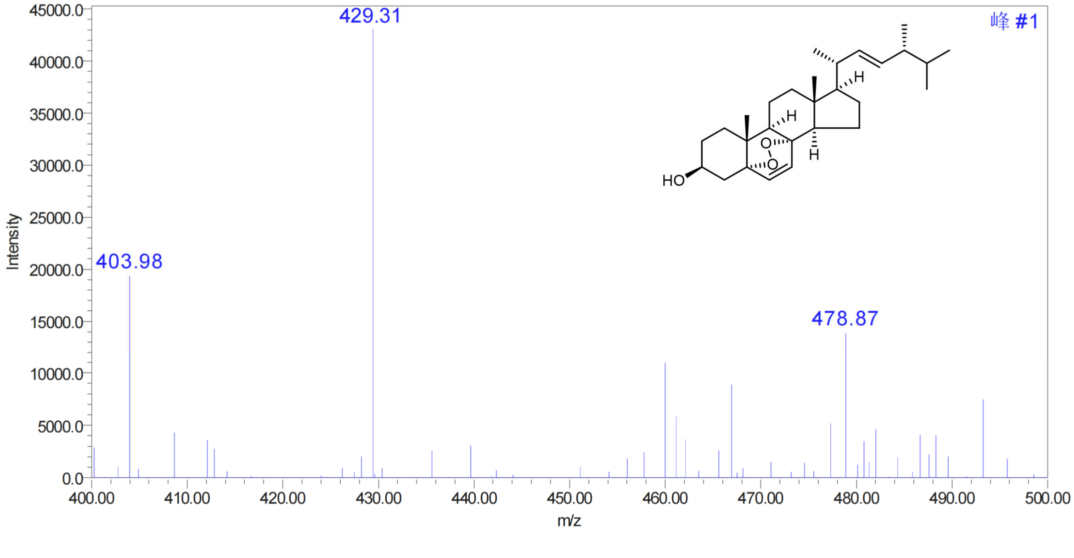

Figure S6. The ESI-MS spectrum of ergosterol peroxide (**2**)

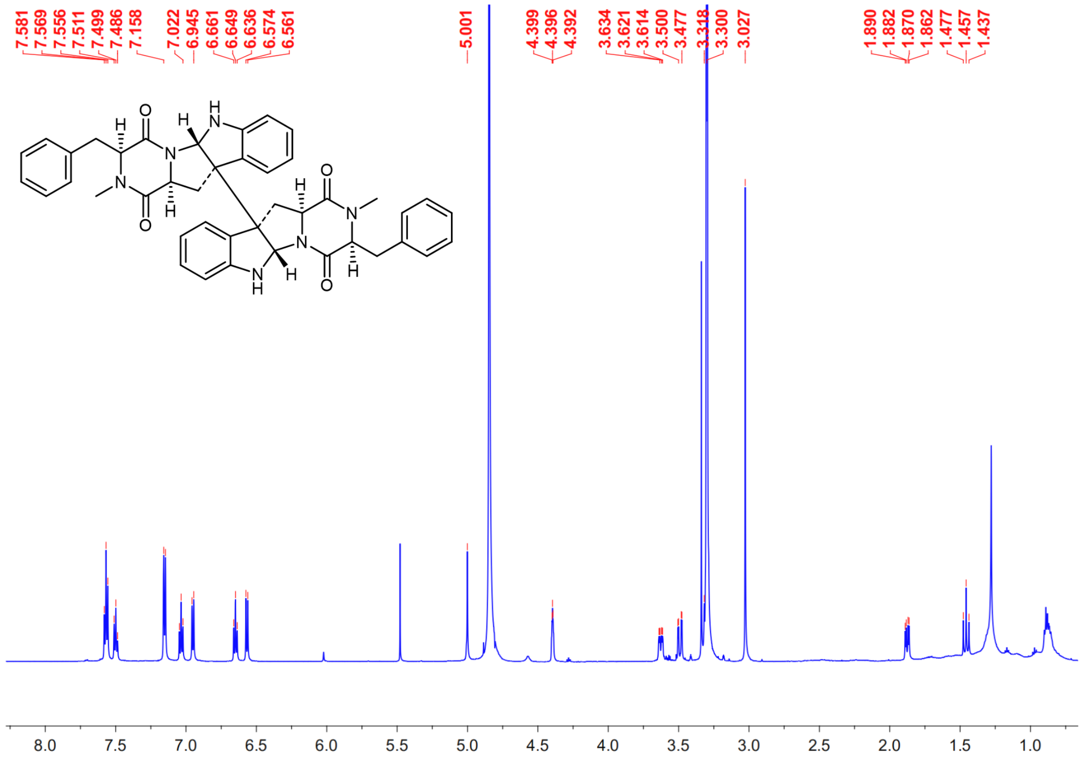

Figure S7. The ¹H-NMR spectrum of (–)-ditryptophenaline (**3**) in CD_3OD

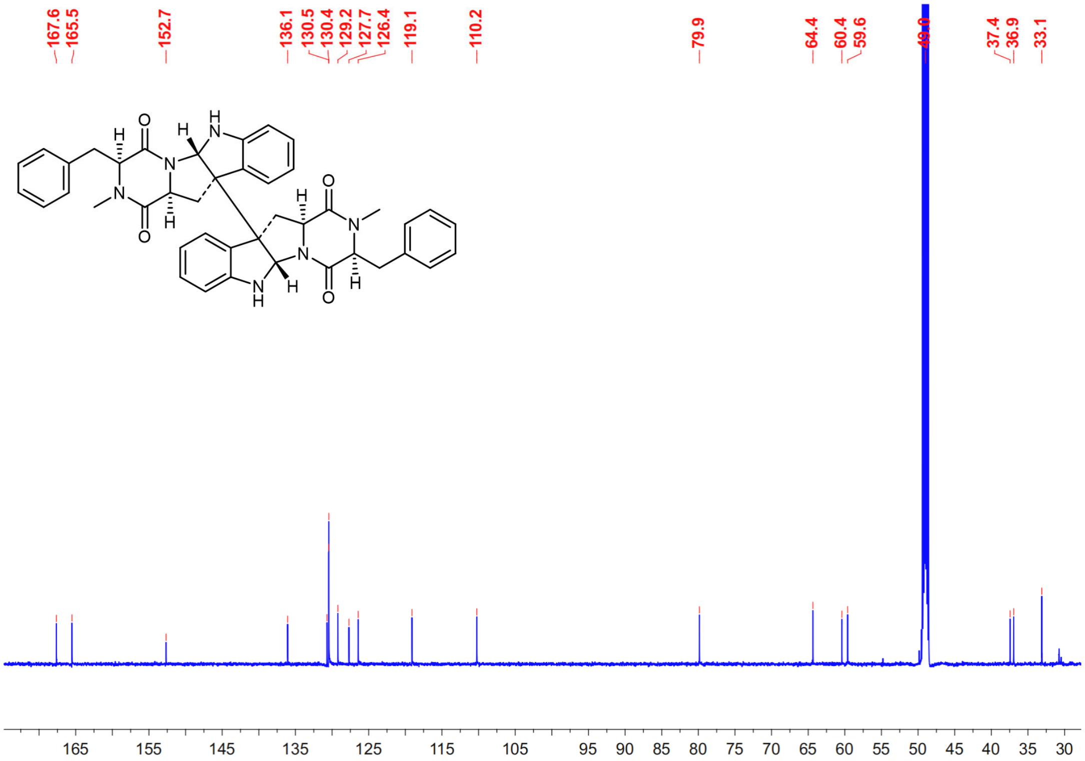

Figure S8. The ¹³C-NMR spectrum of (–)-ditryptophenaline (**3**) in CD_3OD

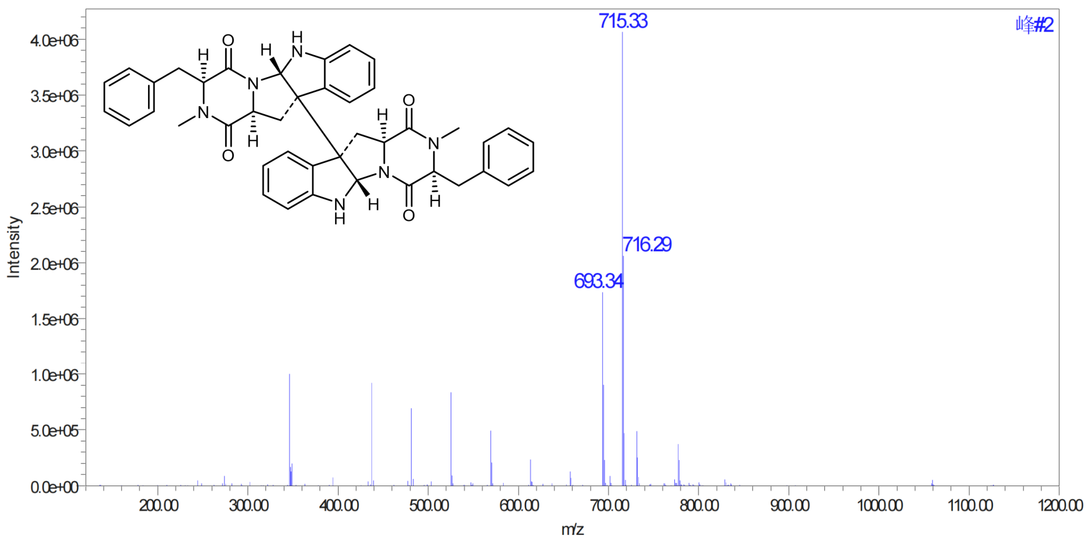

Figure S9. The ESI-MS spectrum of (–)-ditryptophenaline (**3**)

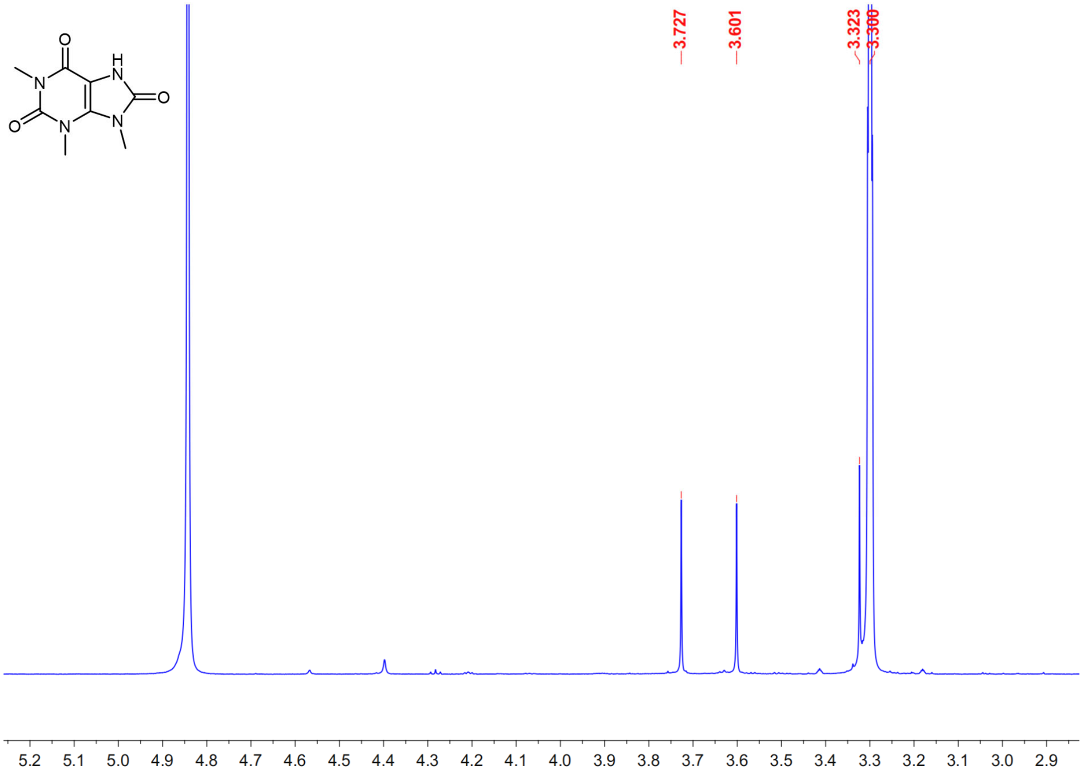

Figure S10. The ^{1}H-NMR spectrum of 1,3,9-trimethyluric acid (**4**) in CD_3OD

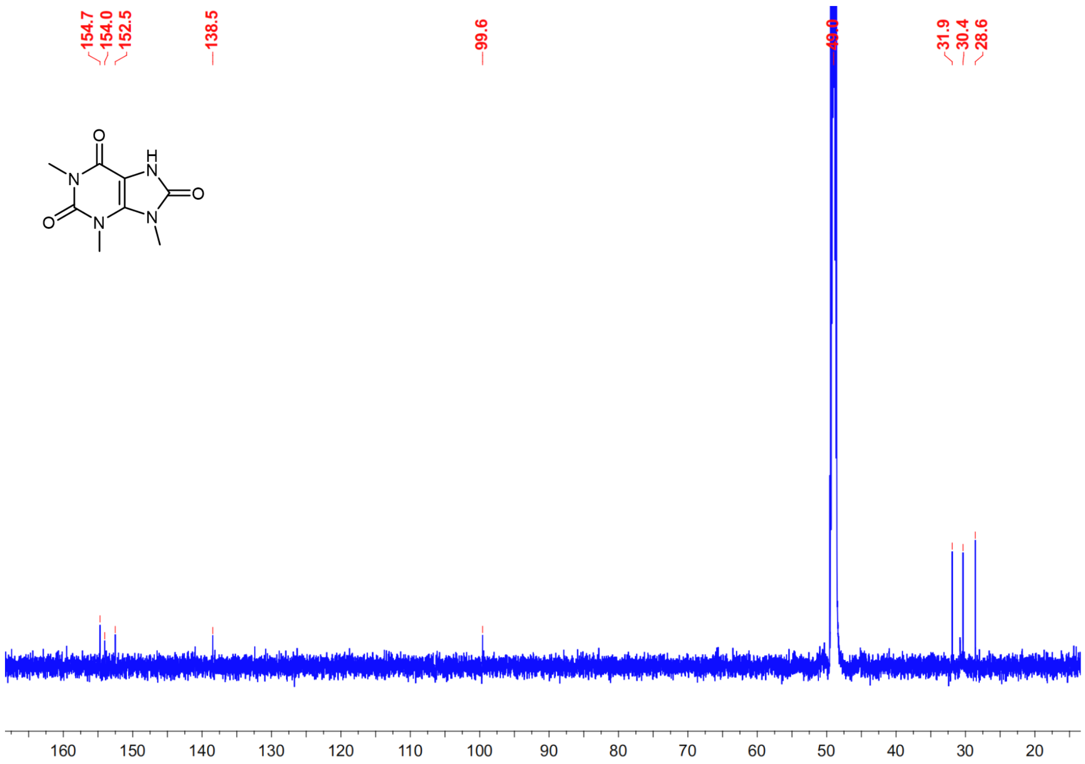

Figure S11. The ^{13}C-NMR spectrum of 1,3,9-trimethyluric acid (**4**) in CD_3OD

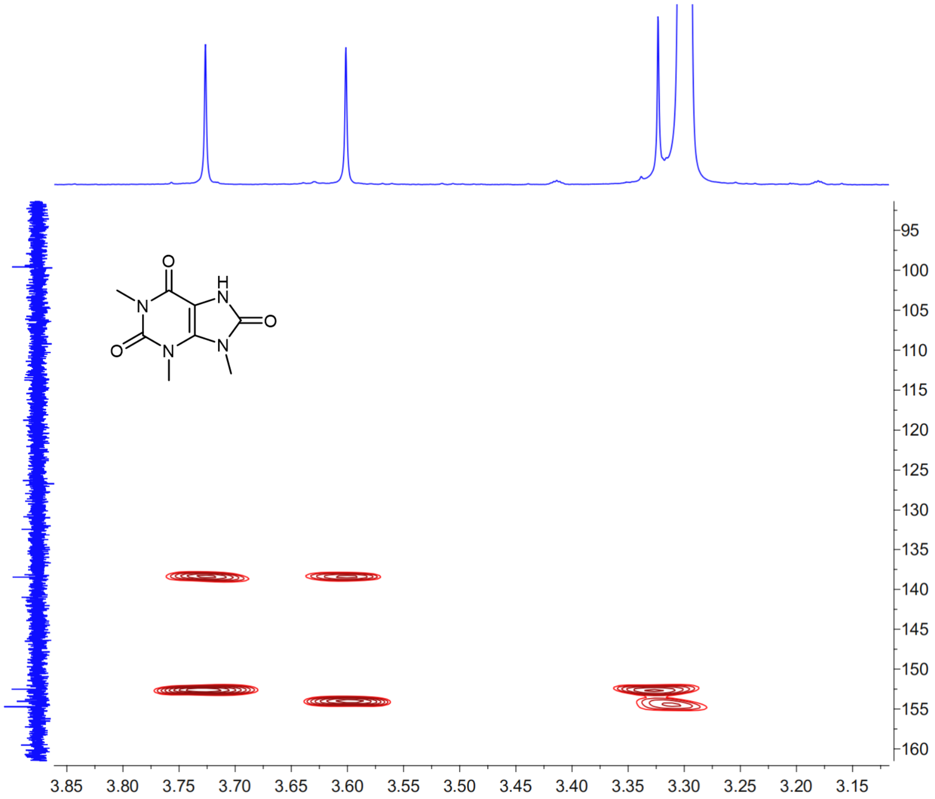

Figure S12. The HMBC spectrum of 1,3,9-trimethyluric acid (**4**) in CD_3OD

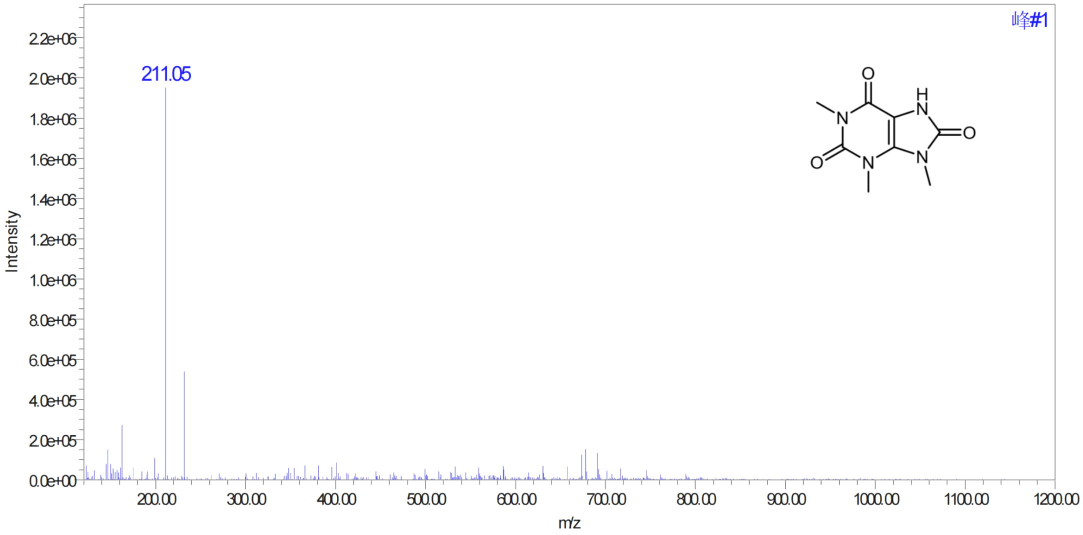

Figure S13. The ESI-MS spectrum of 1,3,9-trimethyluric acid (**4**)

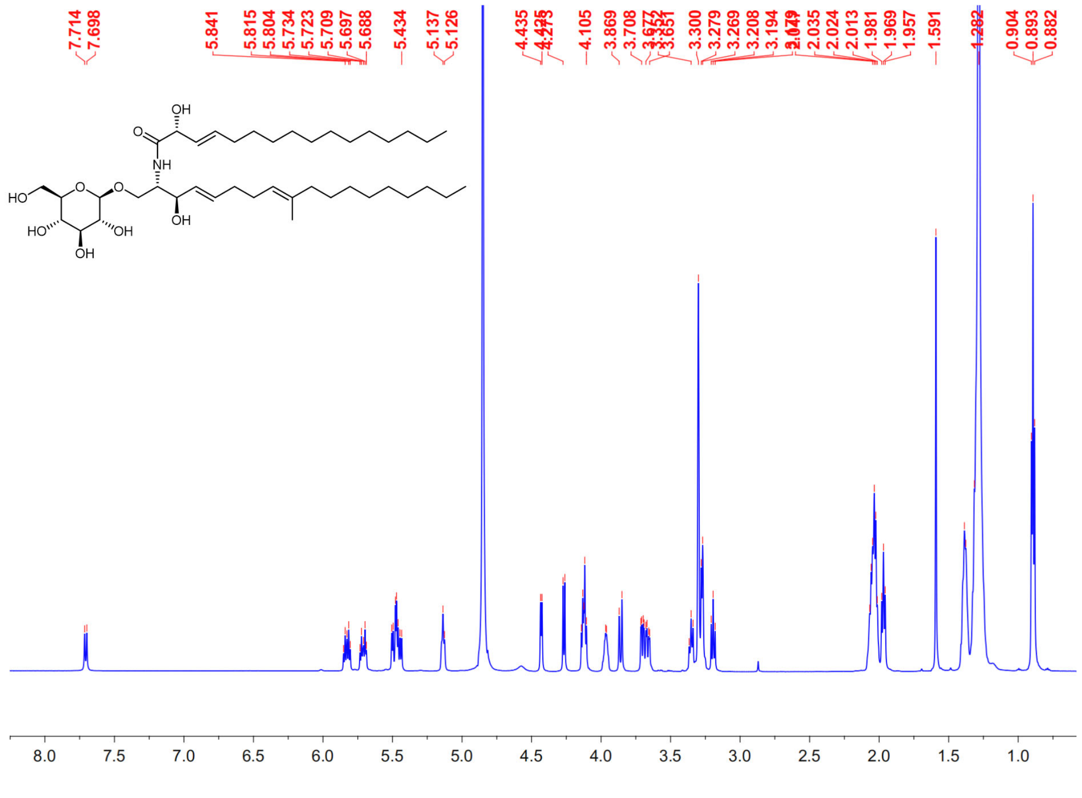

Figure S14. The ^{1}H-NMR spectrum of cerebroside A (**5**) in CD_3OD

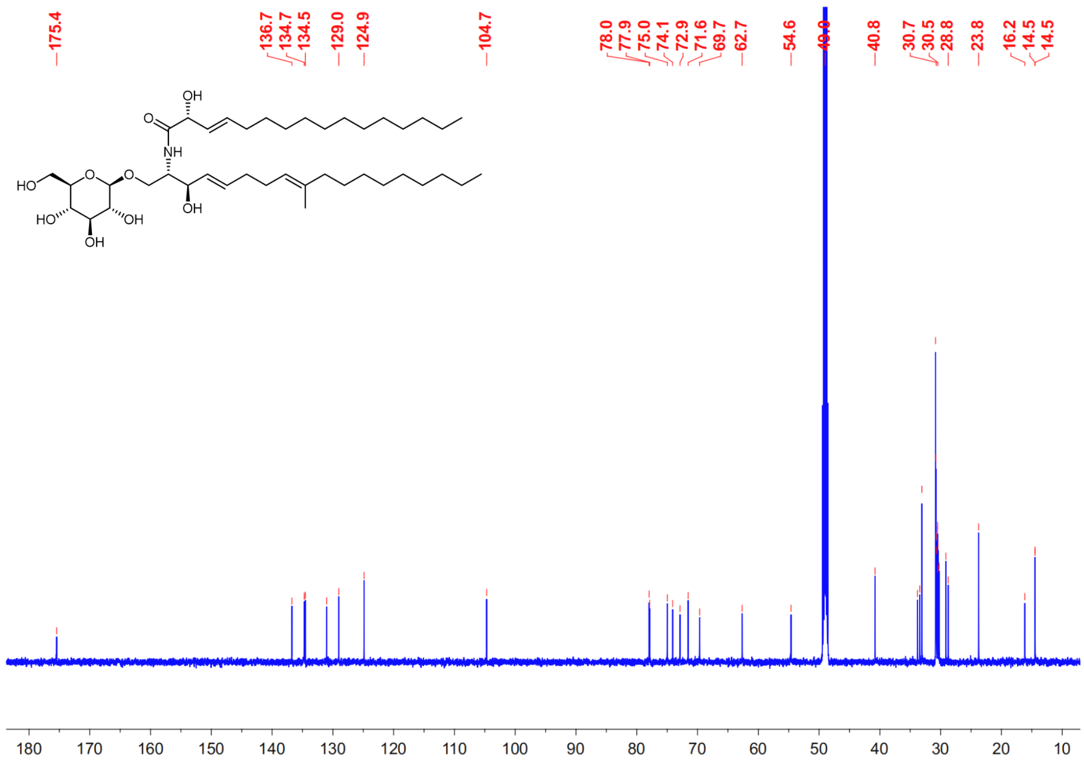

Figure S15. The ^{13}C-NMR spectrum of cerebroside A (**5**) in CD_3OD

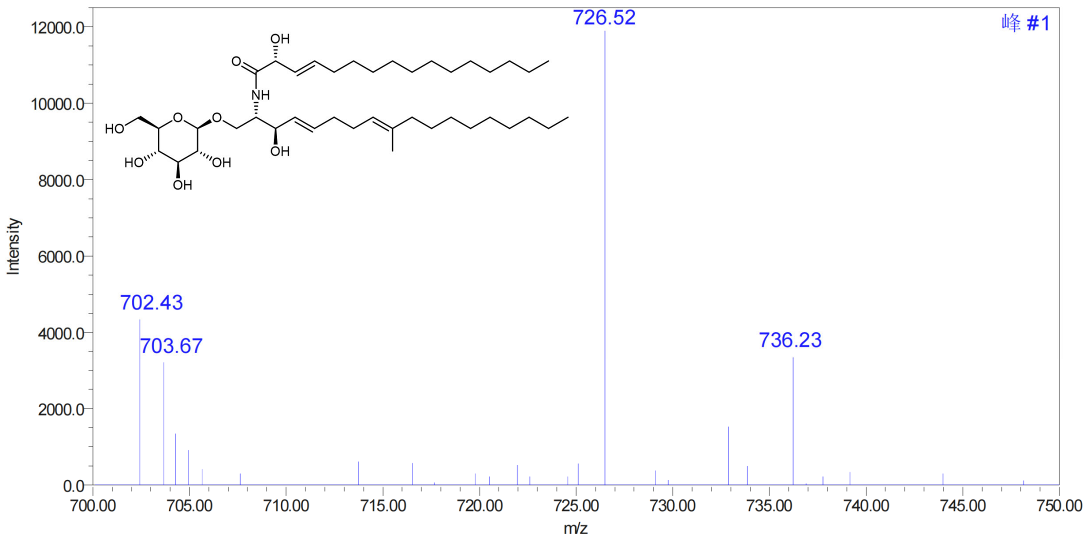

Figure S16. The ESI-MS spectrum of cerebroside A (**5**)

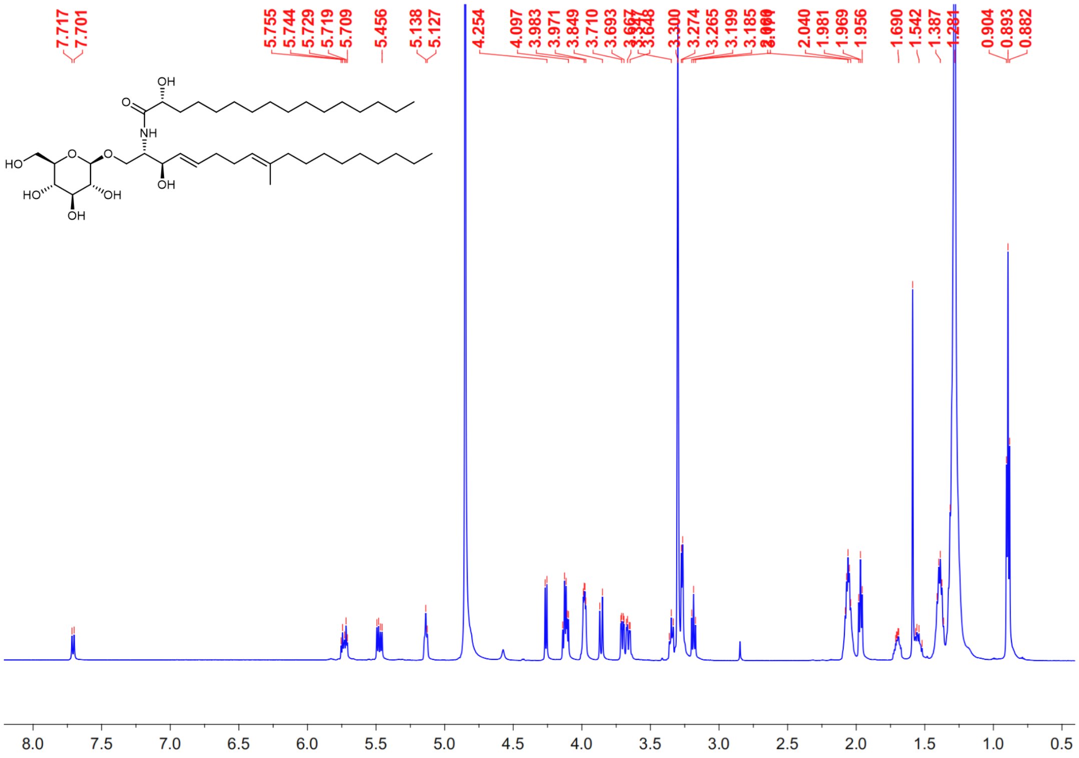

Figure S17. The ^{1}H-NMR spectrum of cerebroside B (**6**) in CD_3OD

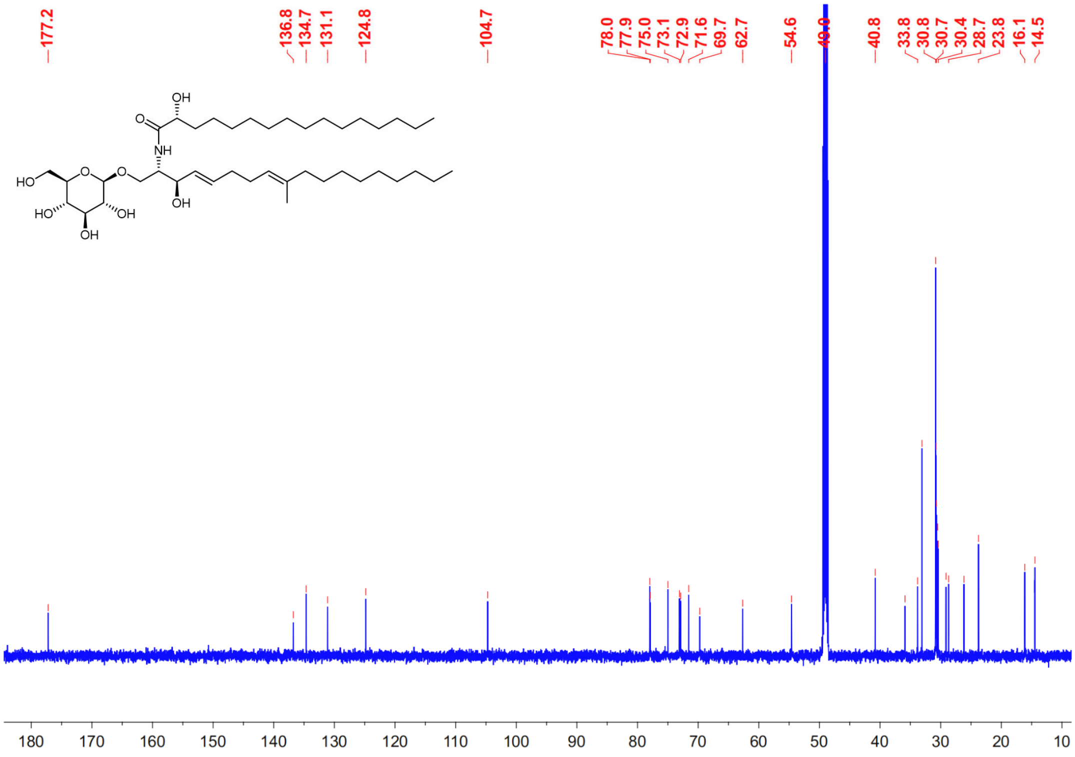

Figure S18. The ^{13}C-NMR spectrum of cerebroside B (**6**) in CD_3OD

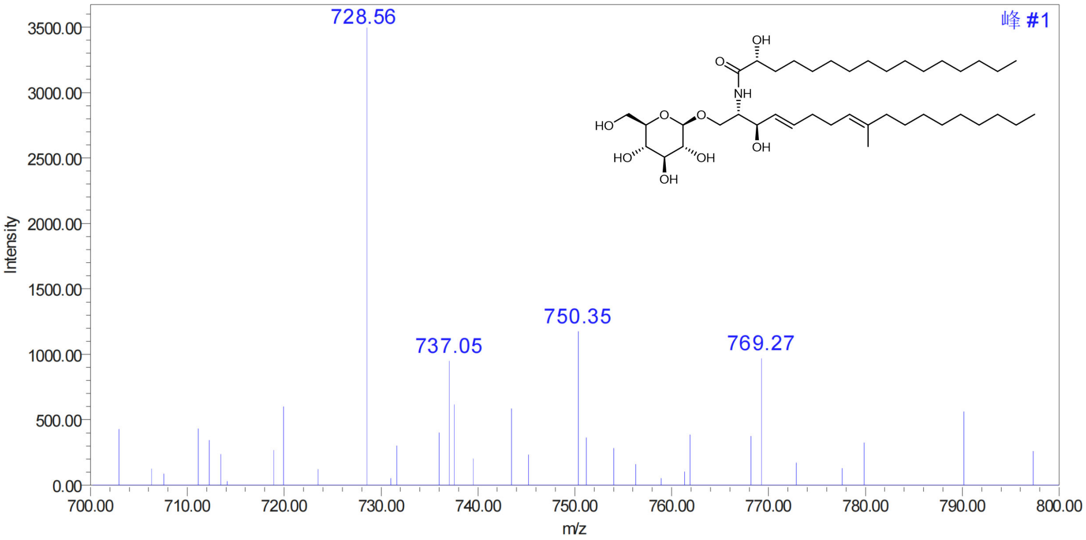

Figure S19. The ESI-MS spectrum of cerebroside B (**6**)

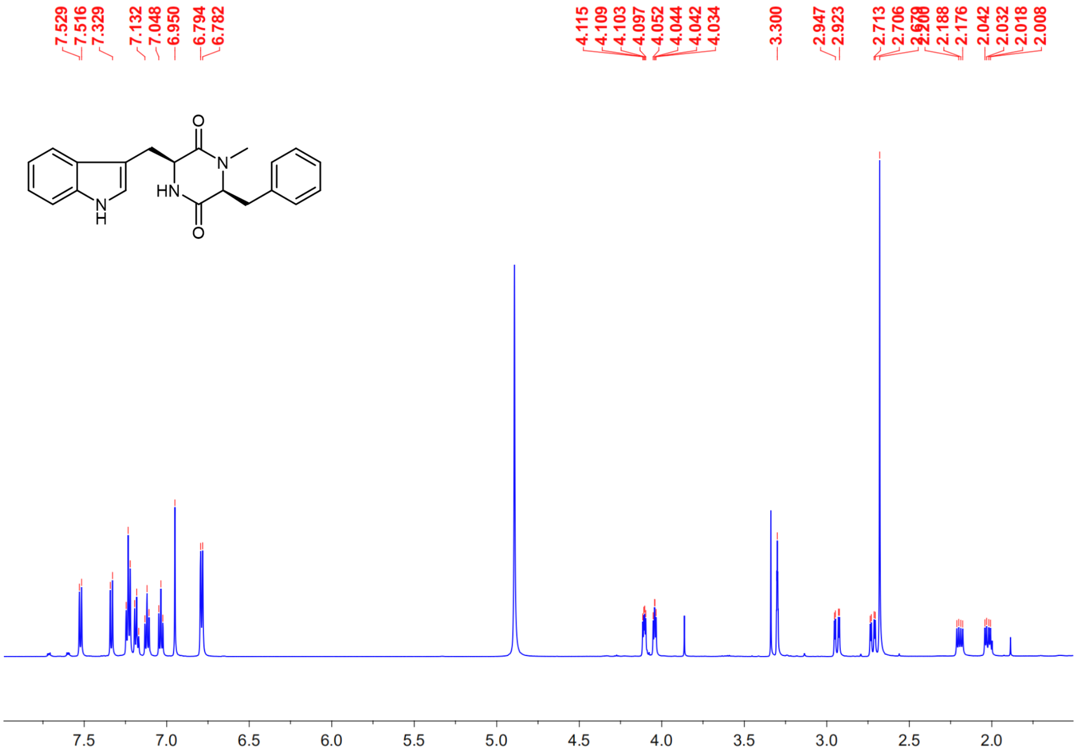

Figure S20. The ^{1}H-NMR spectrum of *cyclo*-*N*-methylphenylalanyltryptophenyl (**7**) in CD_3OD

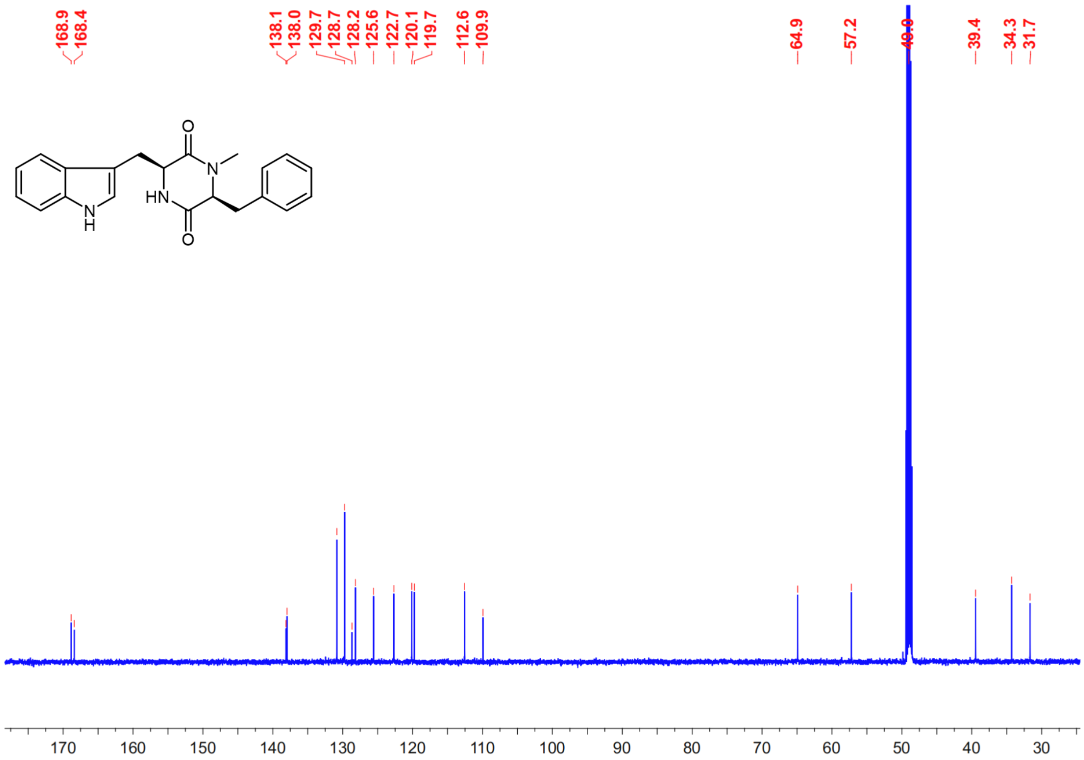

Figure S21. The ^{13}C-NMR spectrum of *cyclo*-*N*-methylphenylalanyltryptophenyl (**7**) in CD_3OD

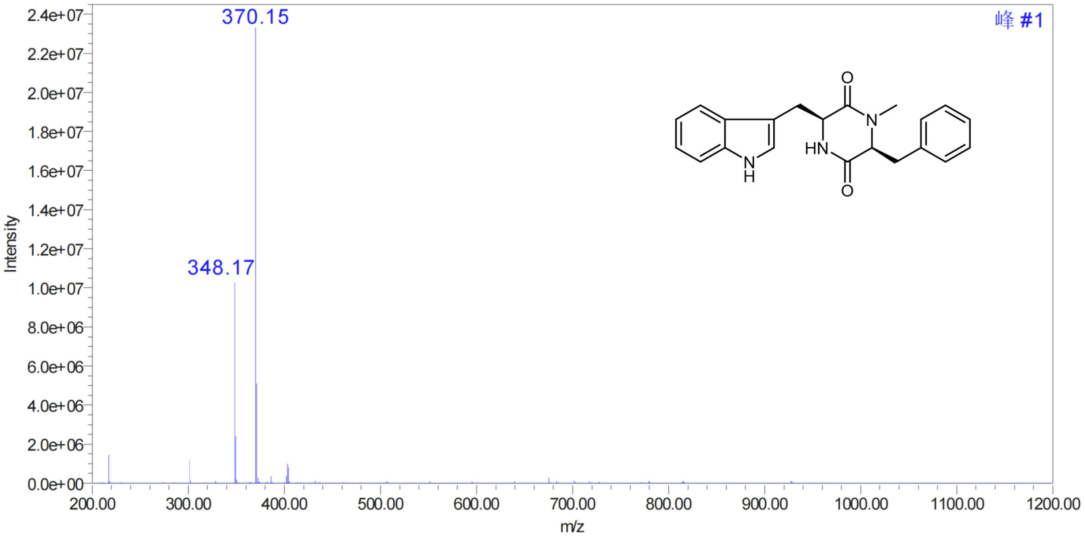

Figure S22. The ESI-MS spectrum of *cyclo*-*N*-methylphenylalanyltryptophenyl (**7**)

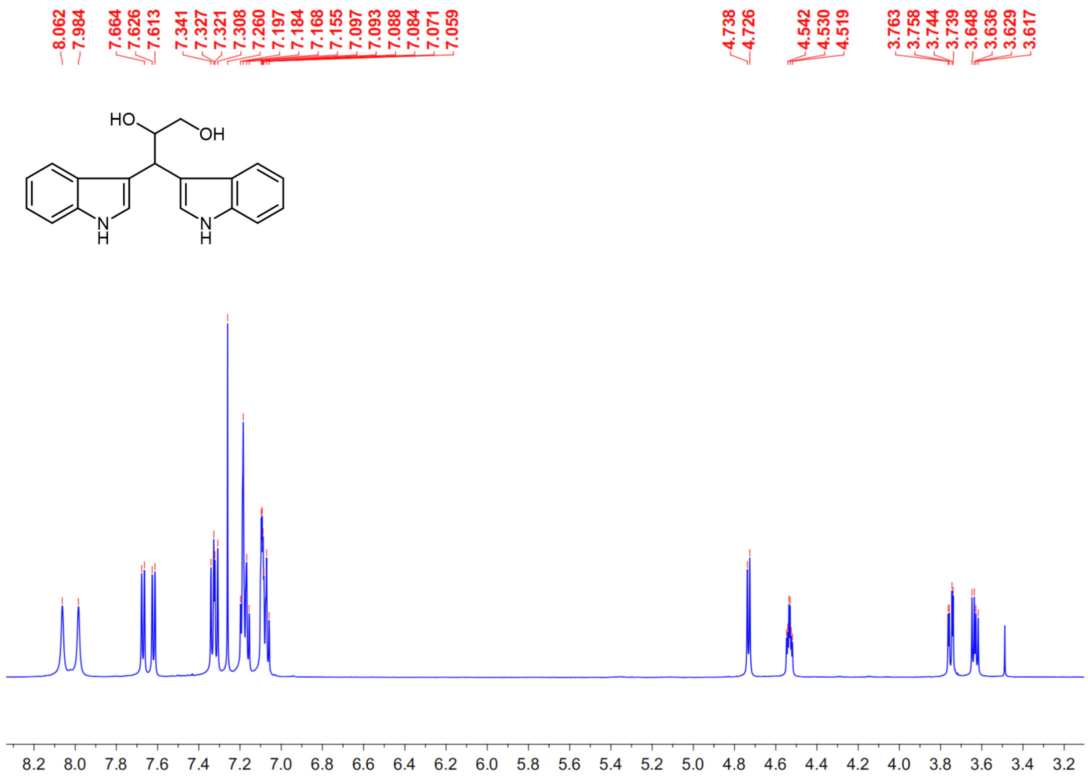

Figure S23. The ^{1}H-NMR spectrum of (2*S*)-3,3-di-1*H*-indol-3-yl-1,2-propanediol (**8**) in $CDCl_3$

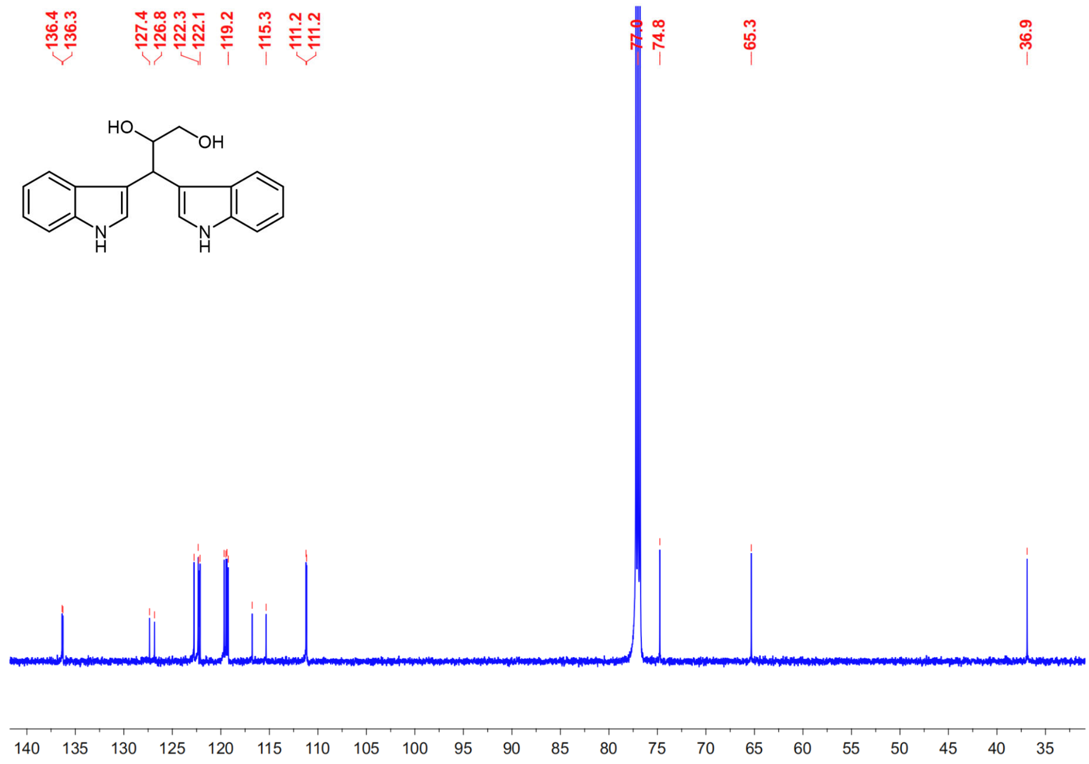

Figure S24. The ^{13}C-NMR spectrum of (2*S*)-3,3-di-1*H*-indol-3-yl-1,2-propanediol (**8**) in $CDCl_3$

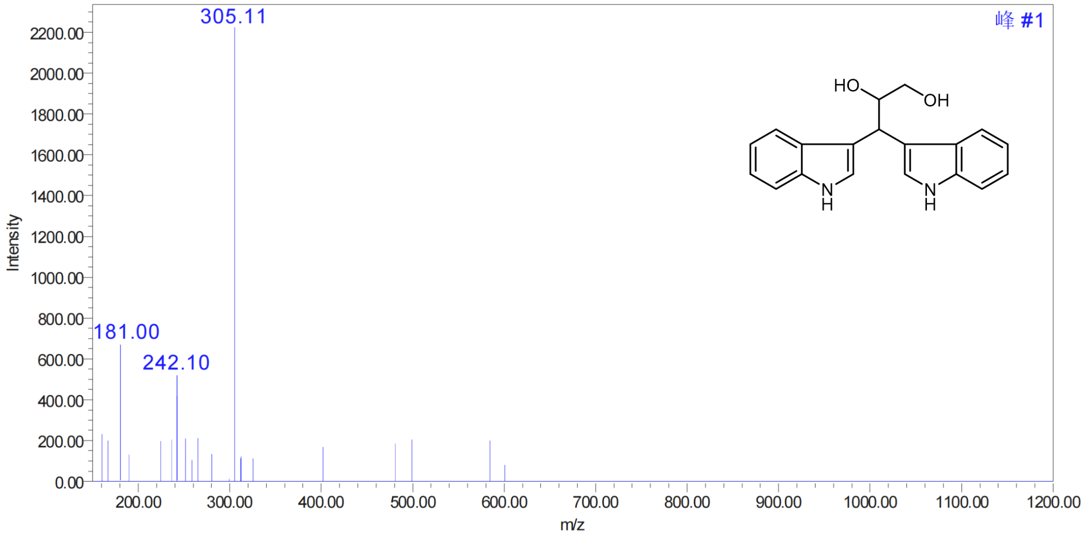

Figure S25. The ESI-MS spectrum of (2*S*)-3,3-di-1*H*-indol-3-yl-1,2-propanediol (**8**)
